# Weighted power summation and contrast normalization mechanisms account for short-latency eye movements to motion and disparity of sine-wave gratings and broadband visual stimuli in humans

**DOI:** 10.1167/jov.24.8.14

**Published:** 2024-08-26

**Authors:** Boris M. Sheliga, Edmond J. FitzGibbon

**Affiliations:** 1 Laboratory of Sensorimotor Research, National Eye Institute, National Institutes of Health, Bethesda, MD, USA

**Keywords:** visual motion, visual disparity, short-latency eye movements

## Abstract

In this paper, we show that the model we proposed earlier to account for the disparity vergence eye movements (disparity vergence responses, or DVRs) in response to horizontal and vertical disparity steps of white noise visual stimuli also provides an excellent description of the short-latency ocular following responses (OFRs) to broadband stimuli in the visual motion domain. In addition, we reanalyzed the data and applied the model to several earlier studies that used sine-wave gratings (single or a combination of two or three gratings) and white noise stimuli. The model provides a very good account of all of these data. The model postulates that the short-latency eye movements—OFRs and DVRs—can be accounted for by the operation of two factors: an excitatory drive, determined by a weighted sum of contributions of stimulus Fourier components, scaled by a global contrast normalization mechanism. The output of the operation of these two factors is then nonlinearly scaled by the total contrast of the stimulus. Despite different roles of disparity (horizontal and vertical) and motion signals in visual scene analyses, the earliest processing stages of these different signals appear to be very similar.

## Introduction

Neurons at the earliest stages of visual processing respond to stimuli over a limited range of spatial and temporal frequencies (for review, see De Valois & De Valois, 1988). However, natural images are typically spatiotemporally broadband and hence, when presented, activate a broad range of spatiotemporal inputs. Therefore, there should exist mechanisms that govern a competition of the inputs of individual neurons and determine how these individual signals interact and are integrated in subsequent processing stages. For example, when a moving stimulus is a sum of two sine-wave gratings of different contrasts, responses are dominated by the higher contrast grating: Small changes in contrast lead to rapid changes in response to that produced by the contrast-dominant grating alone. Such effects have been observed behaviorally (Liu & Sperling, 2006; Matsuura et al., 2008; Sheliga, Kodaka, FitzGibbon, & Miles, 2006b) and in neuronal populations (middle temporal [MT] and middle superior temporal [MST] areas) (Kumbhani, Saber, Majaj, Tailby, & Movshon, 2008; Miura, Inaba, Aoki, & Kawano, 2014b) in the visual motion and in the visual disparity domains (Sheliga, FitzGibbon, & Miles, 2007). Furthermore, certain spatial frequencies (SFs) appear to have more weight than others: Two equal-contrast gratings moving in opposite directions resulted in eye movements in the direction of motion of one of them, even though the response magnitude to each of the gratings presented in isolation was the same (Sheliga et al., 2006b; Sheliga, Quaia, FitzGibbon, & Cumming, 2020).

Over the years, our laboratory has contributed to the advancements in this field of research by using short-latency eye movements as a behavioral response: ocular following responses (OFRs) in the motion domain and disparity vergence responses (DVRs) in the disparity domain. The OFR is a short-latency tracking eye movement evoked by motion of a textured pattern (Gellman, Carl, & Miles, 1990; Miles, Kawano, & Optican, 1986), the characteristics of which reflect activation of the low-level motion detectors involved (for review, see Masson & Perrinet, 2012; Miles, 1998; Miles & Sheliga, 2010). The DVR is a short-latency vergence eye movement evoked by applying small disparities to large textured patterns (Busettini, Fitzgibbon, & Miles, 2001; Busettini, Miles, & Krauzlis, 1996), and many characteristics of DVRs were shown to be consistent with the behavior of disparity-selective neurons in the primate striate cortex (Cumming & DeAngelis, 2001; Cumming & Parker, 1997; Ohzawa, DeAngelis, & Freeman, 1990; Prince, Cumming, & Parker, 2002; Prince, Pointon, Cumming, & Parker, 2002). The extrastriate MT and MST cortical areas were also shown to play a critical role in OFR and DVR generation (Kawano, Inoue, Takemura, Kodaka, & Miles, 2000; Kawano, Shidara, Watanabe, & Yamane, 1994; Takemura, Inoue, & Kawano, 2002; Takemura, Inoue, Kawano, Quaia, & Miles, 2001; Takemura, Kawano, Quaia, & Miles, 2002). In sum, OFRs and DVRs are driven by cortical visual neurons and can be utilized as a behavioral signature of cortical neuronal mechanisms operating at early stages of visual processing.

Sheliga, Quaia, FitzGibbon, and Cumming (2022) set out to evaluate quantitatively the contributions of individual Fourier components of white noise stimuli in generating human DVRs. They proposed a model that fitted the data very well and which posits that the results can be accounted for by the operation of two factors: an excitatory drive, determined by a weighted sum of contributions of stimulus Fourier components, scaled by a global contrast normalization mechanism. DVR amplitudes to broadband stimuli were described by the following equation:

(1) $$\begin{array}{c} \;\text{DVR}=\frac{C^{n}}{C^{n}+C_{50}^{n}}*\frac{{\left[\sum_{i=1}^{N}{\left({\text{DVR}}_{i}*{\left(W_{i}*C_{i}\right)}^{k}\right)}^{m}\right]}^{\frac{1}{m}}}{\sum_{i=1}^{N}{\left(W_{i}*C_{i}\right)}^{k}}\; \end{array}$$

where *DVR_i_* is the response to a given Fourier component presented in isolation. *DVR_i_* is multiplied by the contrast of that component in the image (*C_i_*) and its weight (*W_i_*), normalized by the weighted sum of the contributions of all Fourier components present in the stimulus. This contribution of a single Fourier component is raised to power *k*. The weights of the Fourier components are modeled by a power function of SFs. A competition between contributions of different components is modeled by a power law summation (*m*). *C* is the overall image contrast, and *N* is the number of Fourier components in the image. For pure sine-wave gratings, Equation 1 simplifies to:

(2) $$\begin{array}{c} {\text{DVR}}_{SW}=\frac{C^{m}}{C^{m}+C_{50}^{m}}*{\text{DVR}}_{i}\; \end{array}$$

which is the well-known Naka–Rushton equation (Naka & Rushton, 1966), successfully used to describe DVR contrast dependencies to pure sine-wave stimuli in the past (Quaia, Optican, & Cumming, 2017a; Rambold, Sheliga, & Miles, 2010; Sheliga, FitzGibbon, & Miles, 2006a). *C*_50_ and *n* are, therefore, the Naka–Rushton semi-saturation contrast and power term, respectively.

An analogous model accounted for the results of a study in the visual motion domain (thus, recording the OFRs rather than the DVRs) which used a two-frame, bandpass-filtered, pink noise visual stimuli (Sheliga & FitzGibbon, 2023). The impact of the Fourier component weights in that study was found to be minimal. However, it utilized narrow bandpass stimuli, and the effects of the weights of the components could have been masked by substantial differences in the components’ contrasts. Indeed, modeling the OFRs to stimuli combining two or three gratings that moved in the same or opposite directions did require a weight function, and it was different from the one appropriate for the DVRs: It was the product of a power and an inverted cumulative Gaussian functions (figures 7A and 7H in Sheliga et al., 2020). This suggests that SF-dependent weight functions are different for OFRs and DVRs. However, the Sheliga et al. (2020) model was incomplete, as the contrast response function was not represented explicitly because the measured responses to single gratings at different contrasts were used as inputs to the model.

In this study, we tested the model, formulated by Equation 1, while recording the OFRs to the motion of white noise stimuli and pure gratings. We did not use translational displacements of stimuli but instead adapted stimuli introduced by Quaia, Optican, and Cumming (2017b), who successfully evoked OFRs by shifting the phases of individual Fourier components by a fixed angle. This approach is spatially alias free, as a unidirectional phase shift (1/8th wavelength) is applied to the phases of all the Fourier components in each video frame. Stimuli of three different sizes were used which allowed us to see how the parameters of the model depend on stimulus size. We show that the model provides a very good account of the results. We further applied the model to the data of several earlier OFR and DVR studies which used two or three combined gratings (Quaia et al., 2017b; Sheliga et al., 2007; Sheliga et al., 2006b; Sheliga et al., 2020) and white noise (Sheliga, Quaia, FitzGibbon, & Cumming, 2016) as stimuli. All of these data were well fit by the model.

## Materials and methods

Many of the techniques are described only briefly, as they were similar to those used in this laboratory in the past (e.g., Sheliga, Chen, FitzGibbon, & Miles, 2005). Experimental protocols were approved by the Institutional Review Committee concerned with the use of human subjects. Our research was carried out in accordance with the tenets of the Declaration of Helsinki, and informed consent was obtained for experimentation with human subjects.

### Subjects

Three subjects took part in this study: two were authors (BMS and EJF) and the third was a paid volunteer (JC) naïve as to the purpose of the experiments. All subjects had normal or corrected-to-normal vision. Viewing was binocular.

### Eye-movement recording

The horizontal and vertical positions of the right eye were recorded (sampled at 1 kHz) with an electromagnetic induction technique (Robinson, 1963). A scleral search coil embedded in a silastin ring (Collewijn, Van Der Mark, & Jansen, 1975) was placed in the right eye under topical anesthesia, as described by Yang, FitzGibbon, and Miles (2003). At the beginning of each recording session, a coil calibration procedure was performed using fixation targets monocularly viewed by the right eye.

### Visual display and stimuli

Each subject sat in a darkened room with their head positioned by means of adjustable rests for the forehead and chin and held in place with a head band. Dichoptic stimuli were presented using a Wheatstone mirror stereoscope. Each eye saw a computer monitor (P1230 21-inch cathode ray tube [CRT] monitor; HP Inc., Palo Alto, CA) through a 45° mirror, creating a binocular image 521 mm straight ahead from the corneal vertices of the eyes, which was also the optical distance to the images on the two monitor screens. Thus, the stereoscope was set up for equal vergence and accommodation demand. Each monitor was driven by an independent personal computer (PC; Precision 490; Dell, Round Rock, TX), but the outputs of the video card of each computer (Quadro FX 5600; NVIDIA, Santa Clara, CA) were frame-locked via NVIDIA Quadro G-Sync cards. The monitor screens were each 41.8° wide and 32.0° high and had 1024 × 768-pixel resolution (i.e., 23.4 pixels/° directly ahead of each eye), and the two were synchronously refreshed at a rate of 150 Hz. Each monitor was driven via an attenuator (Pelli, 1997) and a video signal splitter (AC085A-R2; Black Box, Plano, TX), allowing presentation of black-and-white images with an 11-bit equidistant grayscale resolution (mean luminance of 20.8 cd/m^2^). Visual stimuli were seen through a rectangular aperture centered directly ahead of the eyes. The stimuli seen by the two eyes were always the same; we were not sure if we would need binocular manipulations to understand these responses and so opted to use the stereoscope at the outset of the project.

#### Experiment 1

Three types of stimuli were implemented.

##### White noise

**Figure 1.**
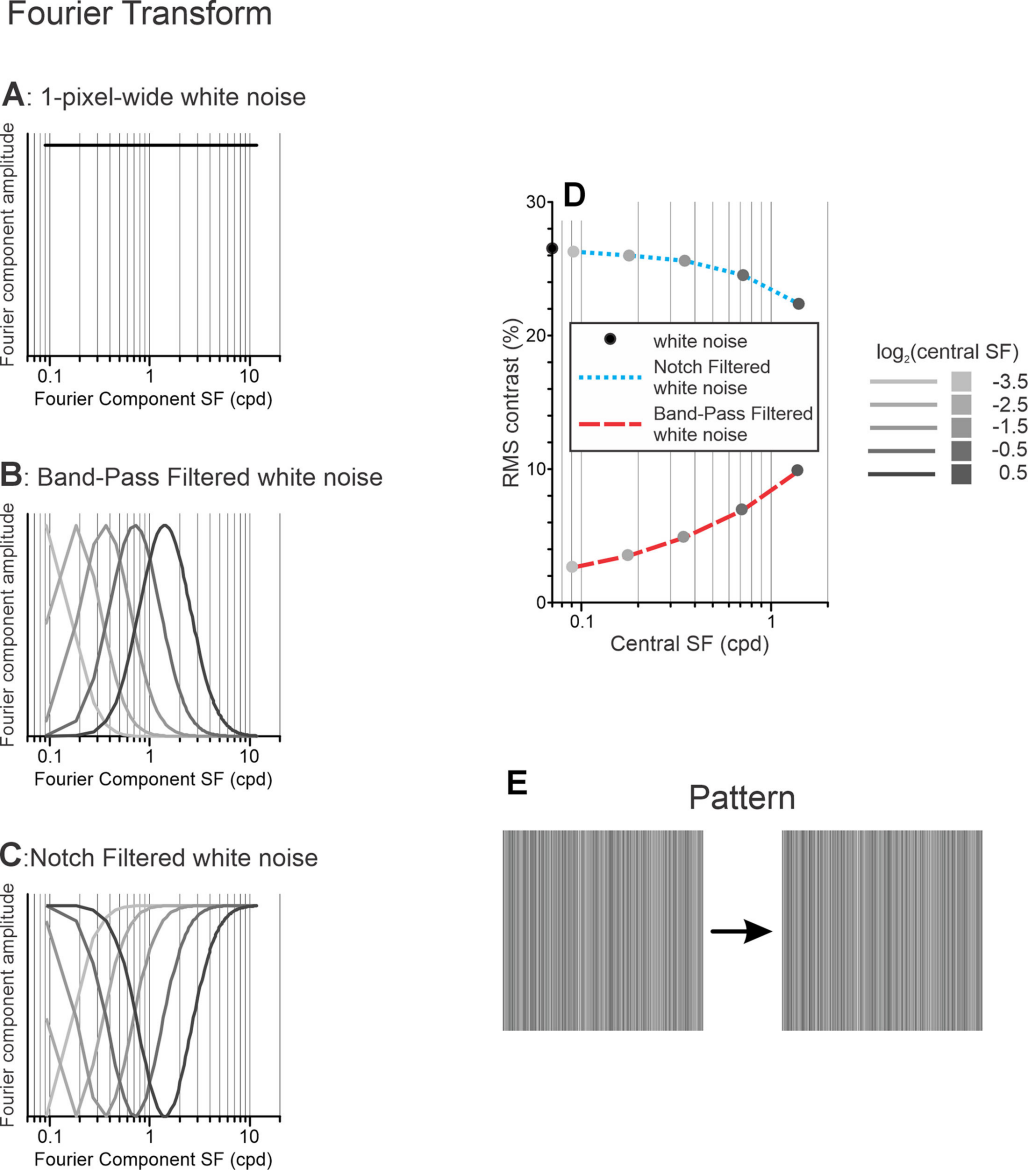
Noise stimuli of Experiment 1. (**A**) Fourier composition of unfiltered white noise. (**B**) log-Gaussian envelopes of filters of bandpass-filtered noise having five different central SFs (grayscale coded; see insert in panel **D**). (**C**) The log-Gaussian envelopes of filters of notch-filtered noise having five different central SFs (grayscale coded; see insert in panel **D**). (**D**) RMS contrast of bandpass-filtered (red dashed line) and notch-filtered (blue dotted line) stimuli having different central SFs (grayscale coded; see insert). (**E**) Scaled versions of 22° × 22° vertical 1D white noise patterns shown in two successive video frames (an example).

Vertical one-dimensional (1D) binary white noise stimuli (random line stimuli [RLS]) were constructed by randomly assigning a black or white value to each successive column of pixels: 1D vertical white noise. The actual luminance values of the black and white pixels were set to 0.35 and 0.65 of maximal luminance, respectively, resulting in 30% root mean square (RMS) contrast. RMS contrast was calculated using the following formula:

(3) $$\begin{array}{c} \text{RMS}=\frac{\sqrt{\frac{\sum_{i=1}^{N}{\left(Lum_{i}-Lum_{mean}\right)}^{2}}{N}}}{Lum_{mean}}\; \end{array}$$

using actual pixel luminance values (*Lum*). One-dimensional fast Fourier transformation (FFT) of a random RLS sample along the horizontal axis—axis of motion—would have quite a lot of variability in the amplitudes of different Fourier components, and those might be very different for another random 1D vertical RLS sample. Such undesirable trial-to-trial variability in the amplitudes of Fourier components would necessarily result in trial-to-trial variability in the magnitude of the OFRs to horizontal motion of these stimuli. We, therefore, calculated the mean of the amplitudes of all Fourier components (in 50 random samples) and set this mean value as the amplitude of each Fourier component of all randomly generated 1D vertical RLS samples used in the experiments reported in this paper (Figure 1A).^1^ This led to a drop of the RMS contrast of all samples from 30% to ∼26.6%. The phases of Fourier components in each sample, on the other hand, remained random.

##### Bandpass-filtered noise

White noise images were filtered using a bandpass filter that was Gaussian on a log scale. The central SFs of the filter varied from 0.0625 to 2 cpd or from 0.088 to 1.414 cpd in half-octave increments for subject BMS^2^ and from 0.088 to 1.414 cpd in octave increments for subjects EJF and JC; the full width at half maximum (FWHM) was always set to 2 octaves. Figure 1B shows FFTs of filtered noise stimuli for five different central SFs. Because the bandwidth was fixed on a log scale, filtered noise stimuli with higher central SFs had higher RMS contrasts (Figure 1D, red dashed line).

##### Notch-filtered noise

A range of SFs was removed from the white noise using the same Gaussian function of SFs as was used for bandpass filtering. Thus, these filters were the complement of the bandpass filters: the sum of the bandpass-filtered image and the notch-filtered image reconstructs the 1D binary white noise pattern. Figure 1C shows FFTs of notch-filtered noise stimuli for five different central SFs. Because the bandwidth of the filter was fixed on a log scale, notch-filtered noise stimuli with higher central SFs had lower RMS contrasts (Figure 1D, blue dotted line).

The horizontal motion of stimuli was not translational. Instead, for each next video frame the phase of each Fourier component shifted by an 1/8th of its respective wavelength, in the same direction for all the Fourier components: forward (i.e., rightward) or backward (i.e., leftward), resulting in 18-3/4–Hz motion temporal frequency (TF). An example of a rightward phase shift is shown in Figure 1E. Note that the appearance of the stimulus changes following such a shift; compare the left and right panels of Figure 1E. The total area covered by visual stimuli could have three different vertical × horizontal dimensions: ∼11° × ∼22° (256 × 512 pixels), ∼22° × ∼11° (512 × 256 pixels), or ∼22° x ∼22° (512 × 512 pixels). A single block of trials had 66 randomly interleaved stimuli: three stimulus types, five central SFs for bandpass- and notch-filtered stimuli, three stimulus sizes, and two signs of the between-frame phase shift (±45°).

#### Experiment 2

Horizontally moving sine-wave gratings had four different SFs (range, 0.088–0.707 cpd in octave increments) and three different RMS contrasts (2.8%, 8.5%, and 22.6%, corresponding to 4%, 12%, and 32% Michelson contrast). Gratings shifted 1/8th wavelength each video frame (i.e., with TF of 18-3/4 Hz). As in Experiment 1, the total area covered by visual stimuli could have three different dimensions: 256 × 512, 512 × 256, or 512 × 512 pixels. A single block of trials had 74 randomly interleaved stimuli: four SFs, three contrasts, three stimulus sizes, two signs of the between-frame phase shift (±45°), plus ±45° phase shift of Fourier components in the 512 × 512-pixel white noise stimulus from Experiment 1, which served as a common condition for the OFR amplitude normalization between Experiments 1 and 2.

### Procedures

Experimental paradigms were controlled by three PCs, which communicated via Ethernet (TCP/IP protocol). The first PC utilized a real-time experimentation software package (REX) (Hays, Richmond, & Optican, 1982), which provided overall control of the experimental protocol, acquisition, display, and storage of the eye-movement data. Two other PCs utilized the Psychophysics Toolbox extensions of MATLAB (MathWorks, Natick, MA) (Brainard, 1997; Pelli, 1997) and generated the visual stimuli.

At the start of each trial a central fixation target (diameter 0.25°) appeared at the center of the otherwise uniform gray (luminance, 20.8 cd/m^2^) screen. To proceed, the subject's eye had to be continuously positioned within 2° of the fixation target for a randomized period of 600 to 1000 ms. The fixation target was then replaced by the first image of a stimulus sequence randomly selected from a lookup table, and one video frame later motion commenced. In 200 ms, the screen turned to uniform gray (luminance, 20.8 cd/m^2^), marking the end of the trial and the start of an intertrial interval. The subjects were asked to refrain from blinking or shifting fixation except during the intertrial intervals but were given no instructions relating to the motion stimuli. A new fixation target appeared after a 500-ms intertrial interval, signaling a new trial. If no saccades were detected (using an eye velocity threshold of 18°/s) for the duration of the trial, then the data were stored; otherwise, the trial was aborted and repeated within the same block. Data collection occurred over several sessions until each condition had been repeated an adequate number of times to permit good resolution of the responses (through averaging); the exact numbers of trials per condition are indicated in the legends of all figures that show experimental data.

### Data analysis

The calibration procedure provided eye position data which were fitted with second-order polynomials and later used to linearize the horizontal eye position data recorded during the experiment. Eye-position signals were then smoothed with an acausal sixth-order Butterworth filter (3 dB at 30 Hz), and mean temporal profiles were computed for each stimulus condition. Trials with microsaccadic intrusions (that had failed to reach the eye-velocity cut-off of 18°/s used during the experiment) were deleted. We utilized position difference measures to minimize the impact of directional asymmetries and boost the signal-to-noise ratio; the mean horizontal eye position with each leftward motion stimulus (45° leftward phase shift in Fourier components) was subtracted from the mean horizontal eye position with the corresponding rightward motion stimulus (45° rightward phase shift in Fourier components). As rightward eye movements were positive in our sign convention, OFRs in the direction of stimulus motion resulted in positive pooled measures. Mean eye velocity was estimated by subtracting position difference measures 10 ms apart (central difference method) and was evaluated every millisecond. Response latency was estimated by determining the time elapsed since the appearance of the second stimulus frame (i.e., the first one in which phases of Fourier components in Experiment 1 or gratings in Experiment 2 appeared shifted) to when the mean eye velocity first exceeded 0.1°/s. The initial OFRs to a given stimulus were quantified by measuring the changes in the mean horizontal eye position signals (OFR amplitude) over the initial open-loop period (i.e., over the period up to twice the minimum response latency). This window always commenced at the same time after the appearance of the second stimulus frame (stimulus-locked measures) and, for a given subject, was the same in Experiments 1 and 2: 68 to 136, 77 to 154, and 67 to 134 ms for BMS, EJF, and JC, respectively. Bootstrapping was used for statistical data evaluation and to construct 68% confidence intervals of the means in the figures (these intervals were smaller than the symbol size in many cases and, therefore, not visible on many graphs).

## Results

### Experiment 1

**Figure 2.**
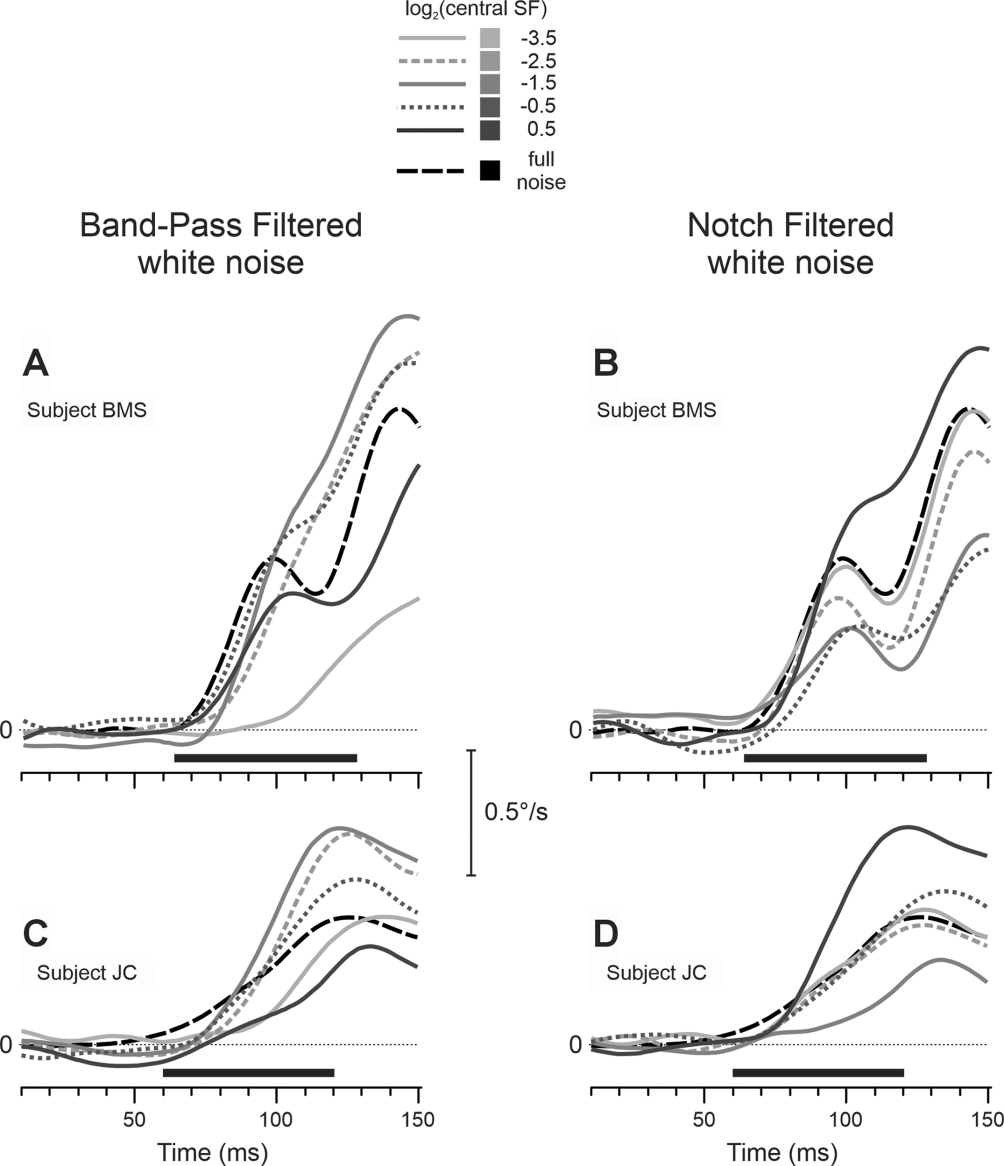
Experiment 1. Mean eye velocity profiles over time to unfiltered white noise (black dashed trace), bandpass-filtered (panels **A** and **C**), and notch-filtered (panels **B** and **D**) noise, whose central spatial frequency is noted by grayscale coding of velocity traces (see the insert). Subjects included BMS (panels **A** and **B**) and JC (panels **C** and **D**). Each trace is the mean response to 130 to 145 (subject BMS) or 92 to 107 (subject JC) repetitions of the stimulus. Abscissa shows the time from the stimulus onset; the horizontal dotted line represents zero velocity; and the horizontal thick black line beneath the traces indicates the response measurement window.

**Figure 3.**
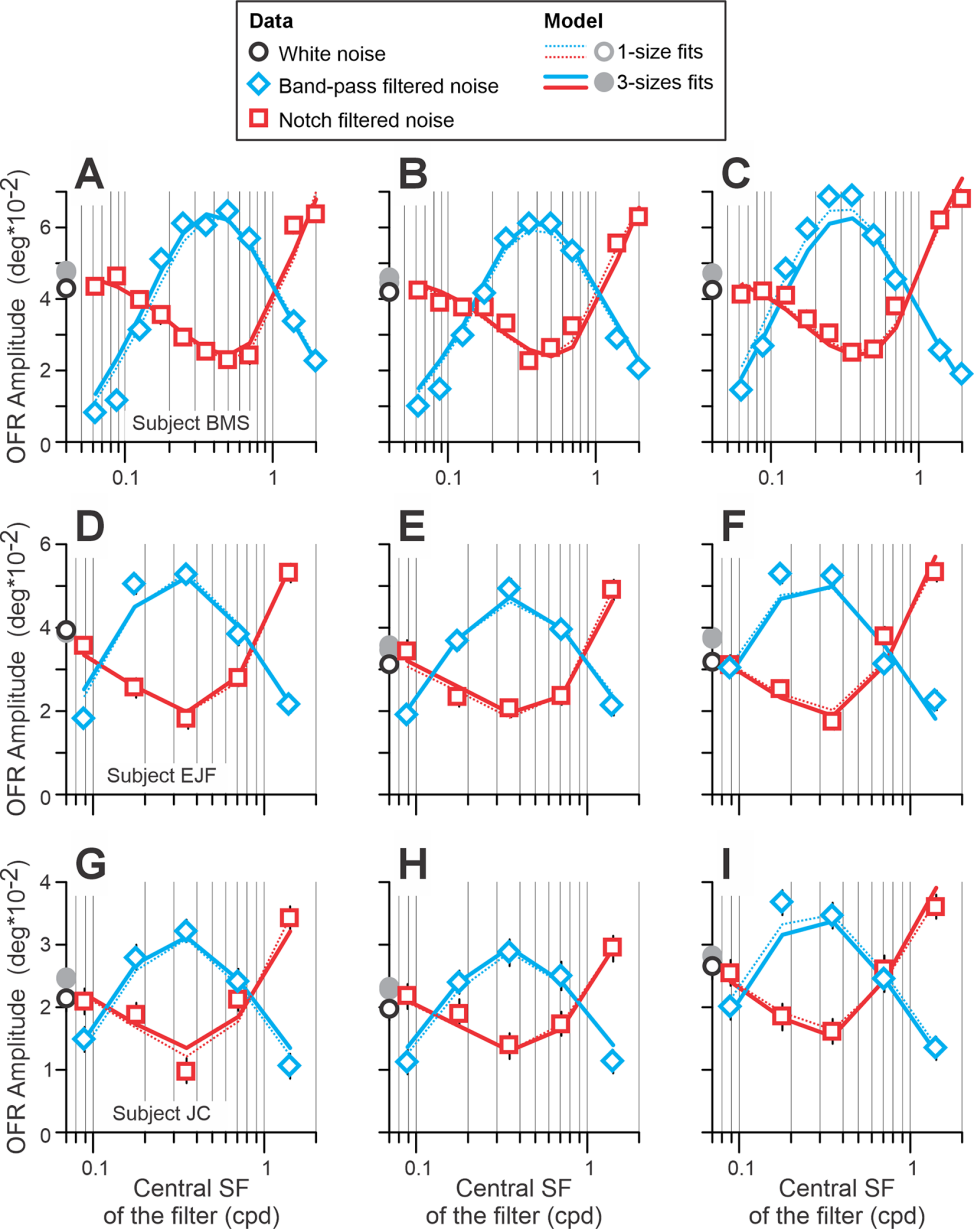
Experiment 1. Dependence of mean OFR amplitude on central SF of the notch filter (data, open red squares; fits, solid red lines), bandpass filter (data, open blue diamonds; fits, solid blue lines), and unfiltered white noise (data, open black circle; fits, filled gray circle). Data for 256 × 512-, 512 × 256-, and 512 × 512-pixel stimuli occupy panels of the left, middle, and right columns, respectively. The data for each subject occupy a single row of panels: subject BMS (panels **A**–**C**; 130–157 trials per condition); subject EJF (panels **D**–**F**; 101–137 trials per condition); subject JC (panels **G**–**I**; 92–107 trials per condition). Equation 4 fits in which the data for each stimulus size were fit separately by eight free parameters are indicated by red (notch-filtered stimuli) and blue (bandpass-filtered stimuli) thin dotted lines.

Figure 2 compares mean eye velocity profiles in response to unfiltered white noise (dashed black trace) with bandpass-filtered (Figures 2A and 2C) and notch-filtered (Figures 2B and 2D) white noise stimuli (noted by grayscale coding of velocity traces) of two subjects—one of the authors (BMS; Figures 2A and 2B) and the naïve subject (JC; Figures 2C and 2D) for the 256 × 512-pixel stimulus. With bandpass-filtered stimuli, the responses were enhanced when central SFs of the filter were within an intermediate range of SFs and reduced when central SFs of the filter were low or high. Conversely, with notch-filtered stimuli, removing intermediate-SF components produced response reductions as the light-gray traces fell below the response to unfiltered noise (dashed trace), whereas removing high-SF components enhanced the OFRs, as dark solid traces lay above the dashed one. These features are quantified in Figure 3, which shows how mean OFR amplitudes changed as a function of the central SF of the bandpass (blue diamonds) and notch (red squares) filters in three subjects (in rows) for 256 × 512-, 512 × 256-, and 512 × 512-pixel stimuli (graphs in the left, middle, and right columns, respectively). The results are very similar for all subjects and all stimulus sizes. For bandpass-filtered stimuli, an enhancement of the OFR amplitude was observed when the central SFs of the filter were between 0.1 and 1 cpd; blue diamonds are located higher than an open black circle (unfiltered white noise condition). Bandpass-filtered stimuli with central SFs beyond this range—higher or lower—produced smaller OFRs; blue diamonds are located lower than an open black circle. For notch-filtered stimuli, a reduction of the OFR amplitude was observed when the central SFs of the filter were between 0.1 and 1 cpd; red squares are located lower than an open black circle. Notch-filtered stimuli with the lowest central SFs produced OFRs similar to unfiltered white noise, whereas those with the highest central SFs produced maximal responses, as red squares are located much higher than an open black circle. A visual inspection of the data also reveals that peaks (bandpass-filtered stimuli) and troughs (notch-filtered stimuli) for 512 × 512-pixel stimuli in the right column graphs of Figure 3 are shifted slightly toward lower SFs than peaks and troughs for smaller stimulus sizes.

### Experiment 2

**Figure 4.**
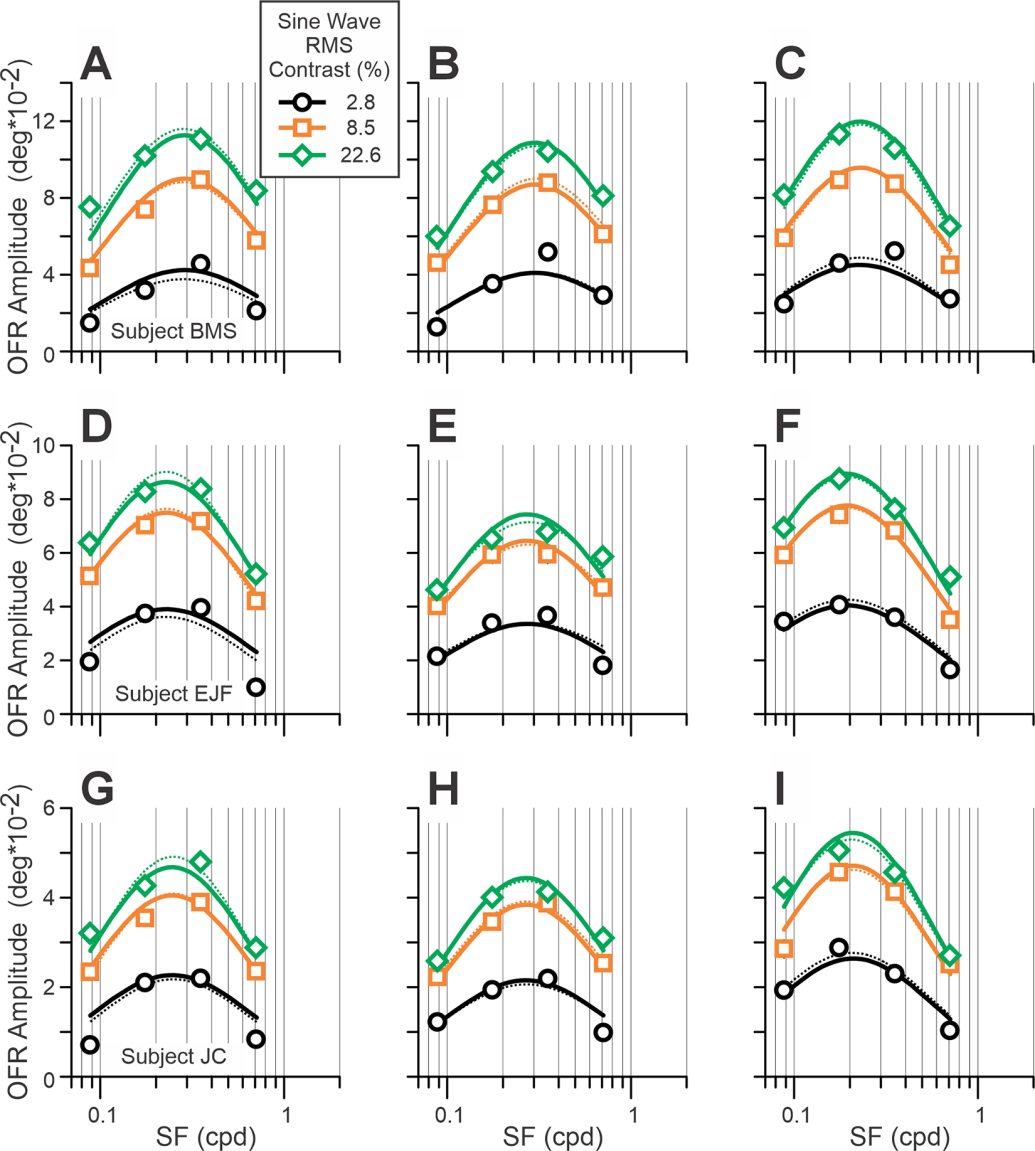
Experiment 2. Dependence of mean OFR amplitude on SF of single gratings (data, symbols; fits, solid lines). The data for gratings of different contrast are symbol and color coded (see rectangular insert). Data for 256 × 512-, 512 × 256-, and 512 × 512-pixel stimuli occupy panels of the left, middle, and right columns, respectively. The data for each subject occupy a single row of panels: subject BMS (panels **A**–**C**; 123–137 trials per condition); subject EJF (panels **D**–**F**; 84–122 trials per condition); subject JC (panels **G**–**I**; 105–122 trials per condition). Equation 4 fits in which the data for each stimulus size were fit separately by eight free parameters are shown by thin dotted lines, color-coded for stimulus contrast.

Figure 4 shows data of three subjects (in rows) for the OFR SF tuning for horizontally moving 1D vertical sine-wave gratings with 256 × 512-, 512 × 256-, and 512 × 512-pixel stimuli (graphs in the left, middle, and right columns, respectively). The data for gratings of different contrast are color and symbol coded (see rectangular insert). As would be expected, the amplitude of the OFRs increased with the contrast of the grating. For a given stimulus size, the responses appeared to peak at similar SFs. However, the peak responses are visibly shifted toward lower SFs for 512 × 512 stimuli compared to the other two stimulus sizes. All SF dependences were very well fit by Gaussian functions of log SF (median *r*^2^ = 0.986; range, 0.921–1.000; not shown).

### Model

The data of Experiments 1 and 2 were fit by Equation 1, although in this study the OFRs are being modeled; that is,

(4) $$\begin{array}{c} \;\text{OFR}=\frac{C^{n}}{C^{n}+C_{50}^{n}}*\frac{{\left[\sum_{i=1}^{N}{\left({\text{OFR}}_{i}*{\left(W_{i}*C_{i}\right)}^{k}\right)}^{m}\right]}^{\frac{1}{m}}}{\sum_{i=1}^{N}{\left(W_{i}*C_{i}\right)}^{k}}\; \end{array}$$

where *OFR_i_* is the response to a given Fourier component, derived from the log-Gaussian fit to the OFR SF tuning:

(41) $$\begin{array}{c} \text{OFR}\left({\text{SF}}_{i}\right)=A_{MAX}*e^{-\frac{{\left[\log_{2}\left({\text{SF}}_{i}\right)-\log_{2}\left(\mu_{SF}\right)\right]}^{2}}{2*\sigma_{SF}^{2}}}\; \end{array}$$

where *SF_i_* is a spatial frequency of this Fourier component, and *A_MAX_*, µ*_SF_*, and σ*_SF_* are the first three free parameters of the model. *OFR_i_* is multiplied by the contrast of that component in the image (*C_i_*) and its weight (*W_i_*), normalized by the weighted sum of the contributions of all Fourier components present in the stimulus. A single Fourier component contribution is raised to the power *k*, the fourth free parameter of the model. A competition between contributions of different components is modeled by a power-law summation, *m* being the fifth free parameter of the model. *C* is the overall RMS stimulus contrast, and *N* is the number of Fourier components in the image (128 for 512 × 256-pixel stimuli; 256 for 256 × 512- and 512 × 512-pixel ones). The weights of the Fourier components were modeled by an inverted cumulative Gaussian function of log SF:

(42) $$\begin{array}{c} \;W\left({\text{SF}}_{i}\right)=1-\frac{1}{2}*\left[1+erf\left(\frac{\log_{2}{\text{SF}}_{i}-\log_{2}\left(\mu_{W}\right)}{sqrt\left(2\right)*\sigma_{W}}\right)\right]\; \end{array}$$

where µ*_W_* and σ*_W_* are the sixth and seventh free parameters of the model. This function lacks a scaling free parameter (it is set to 1), because the *W_i_* values appear in both the numerator and the denominator of Equation 4; therefore, any scaling coefficient is canceled out. *C*_50_ and *n* are the last two (eighth and ninth) free parameters of the model. For pure sine-wave gratings, Equation 4 simplifies to

(4c) $$\begin{array}{c} {\text{OFR}}_{SW}={\text{OFR}}_{SW(MAX)}*\frac{C^{n}}{C^{n}+C_{50}^{n}}\; \end{array}$$

which is the Naka–Rushton equation (Naka & Rushton, 1966) used to describe OFR contrast dependencies to pure sine-wave stimuli in the past (Barthelemy, Perrinet, Castet, & Masson, 2008; Miura et al,. 2006; Quaia et al., 2017b; Quaia, Optican, & Cumming, 2018; Quaia, Sheliga, Fitzgibbon, & Optican, 2012; Sheliga et al., 2005; Sheliga, Quaia, FitzGibbon, & Cumming, 2013). Thus, *C*_50_ and *n* are the Naka–Rushton semi-saturation contrast and power term, respectively. *OFR_SW(MAX)_* is the maximal attainable response for the sine wave of a given SF, calculated using Equation 4a.

**Figure 5.**
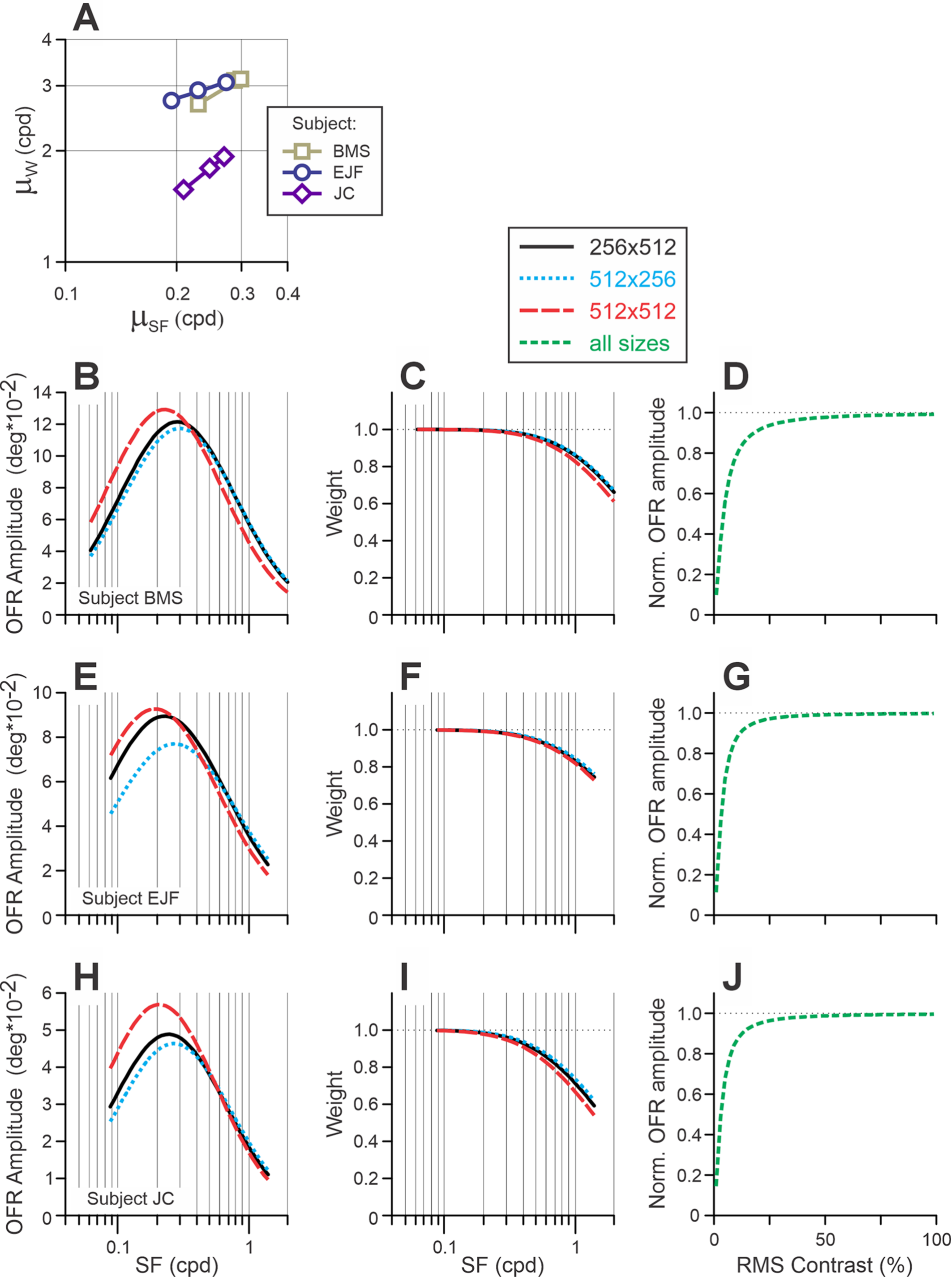
Model. (**A**) Linear relationship (on log/log scale) between the best-fit µ*_SF_* and µ*_W_* free parameters. Data of different subjects are symbol and color coded (see rectangular insert). (**B**–**J**) The relationships between stimulus parameters and the model fits for each subject (in rows). (**B**, **E**, **H**) Equation 4a, OFR SF tuning; (**C**, **F**, **I**) Equation 4b, the function relating SF of the Fourier components to *W*; (**D**, **G**, **J**) Equation 4c, normalized Naka–Rushton equation. Fits for different sizes are color coded (see rectangular insert).

Equation 4 provided very good fits to the data for each stimulus size in all subjects (median *r*^2^ = 0.970; range, 0.948–0.977). Analyzing the values of the best-fit free parameters, we noticed that σ*_SF_* was similar to σ*_W_* for any given stimulus size in each subject. Constraining σ*_SF_* and σ*_W_* to be the same caused minimal deterioration of the fits (median, –0.02%; range, –0.09% to 0.00%) and was not statistically significant (general linear *F*-test). The resulting fits (eight free parameters for each stimulus size) are indicated in Figure 3 for Experiment 1 and in Figure 4 for Experiment 2 by thin dotted lines. Sheliga et al. (2020) modeled OFRs to stimuli combining two or three gratings and showed that responses to stimuli of two different sizes can be successfully fit using a single set of free parameters, except for µ*_SF_* and µ*_W_*, which did change with size. We did the same here, fitting the data for all three stimulus sizes in Experiments 1 and 2 using a single set of free parameters, except for µ*_SF_*, µ*_W_*, and *A_MAX_*. *A_MAX_* was added to this list, because we did not have a quantitative hypothesis as to how *A_MAX_* should change with stimulus size. Thus, there were 14 free parameters, in total. The resulting fits were good (*r*^2^ = 0.965, 0.964, and 0.953 for subjects BMS, EJF, and JC, respectively). Analyzing the values of the best-fit free parameters, we noticed that the best-fit µ*_SF_* and µ*_W_* appeared to be related (Figure 5A, symbols). We attempted a linear fit on a log/log scale (i.e., using a minimal number of free parameters: 2):

(5) $$\begin{array}{c} \log_{2}\;\mu_{W}=A*\log_{2}\;\mu_{SF}+B\; \end{array}$$

The fits were very good (*r*^2^ = 0.991, 0.990, and 1.000 for subjects BMS, EJF, and JC, respectively) (Figure 5A, lines). Thus, the data fits now had 13 free parameters (*A* and *B* from Equation 5) instead of three separate µ*_W_* free parameters for each stimulus size. The goodness of fits was barely changed (*r*^2^ = 0.965, 0.964, and 0.953 for subjects BMS, EJF, and JC, respectively). Total deterioration of fits—due to a reduction in the number of free parameters from 27 (9 free parameters × 3 stimulus sizes) to 13—was not statistically significant: general linear *F*-test *F*(14, 72) = 1.42, *F*(14, 42) = 0.71, and *F*(14, 42) = 0.77 for subjects BMS, EJF, and JC, respectively. These fits are indicated in Figure 3 for Experiment 1 and in Figure 4 for Experiment 2 by thick solid lines, and Table 1 lists the best-fit values of free parameters. Figures 5B to 5J presents best-fit OFR SF tuning functions (Figures 5B, 5E, and 5H; Equation 4a), weight functions (Figures 5C, 5F, and 5I; Equation 4b), and normalized Naka–Rushton functions (Figures 5D, 5G, and 5J; Equation 4c) for three subjects.

**Table 1.** Best-fit parameters of Equation 4 fits.

									*Linear fit*		
Subject	Stimulus size (*V_pix_* × *H_pix_*)	*AMAX* (°)	µ*_SF_* (cpd)	σ*_SF_* and σ*_W_* (log_2_ units)	*C*_50_	*n_C_*	*k*	*m*	*A*	*B*	*r* ^2^
BMS	256 × 512	0.12	0.287	1.49	0.04	1.5	2.0	0.97	0.61	2.7	0.965
	512 × 256	0.12	0.299								
	512 × 512	0.13	0.229								
EJF	256 × 512	0.09	0.229	1.59	0.03	1.7	2.7	0.99	0.34	2.3	0.964
	512 × 256	0.08	0.273								
	512 × 512	0.09	0.194								
JC	256 × 512	0.05	0.246	1.46	0.03	1.6	1.8	0.95	0.81	2.5	0.953
	512 × 256	0.05	0.269								
	512 × 512	0.06	0.209								

In all three subjects, the best-fit µ*_SF_* values were the lowest for 512 × 512-pixel stimuli, intermediate for 256 × 512-pixel stimuli, and highest for 512 × 256-pixel stimuli (Figures 5B, 5E, and 5H and Table 1). The difference between 512 × 512-pixel stimuli and stimuli of two other sizes was statistically significant in all subjects (*p* < 0.001), but the difference in the best-fit µ*_SF_* values between 256 × 512-pixel versus 512 × 256-pixel stimuli was significant only for subject EJF (*p* < 0.001).

**Figure 6.**
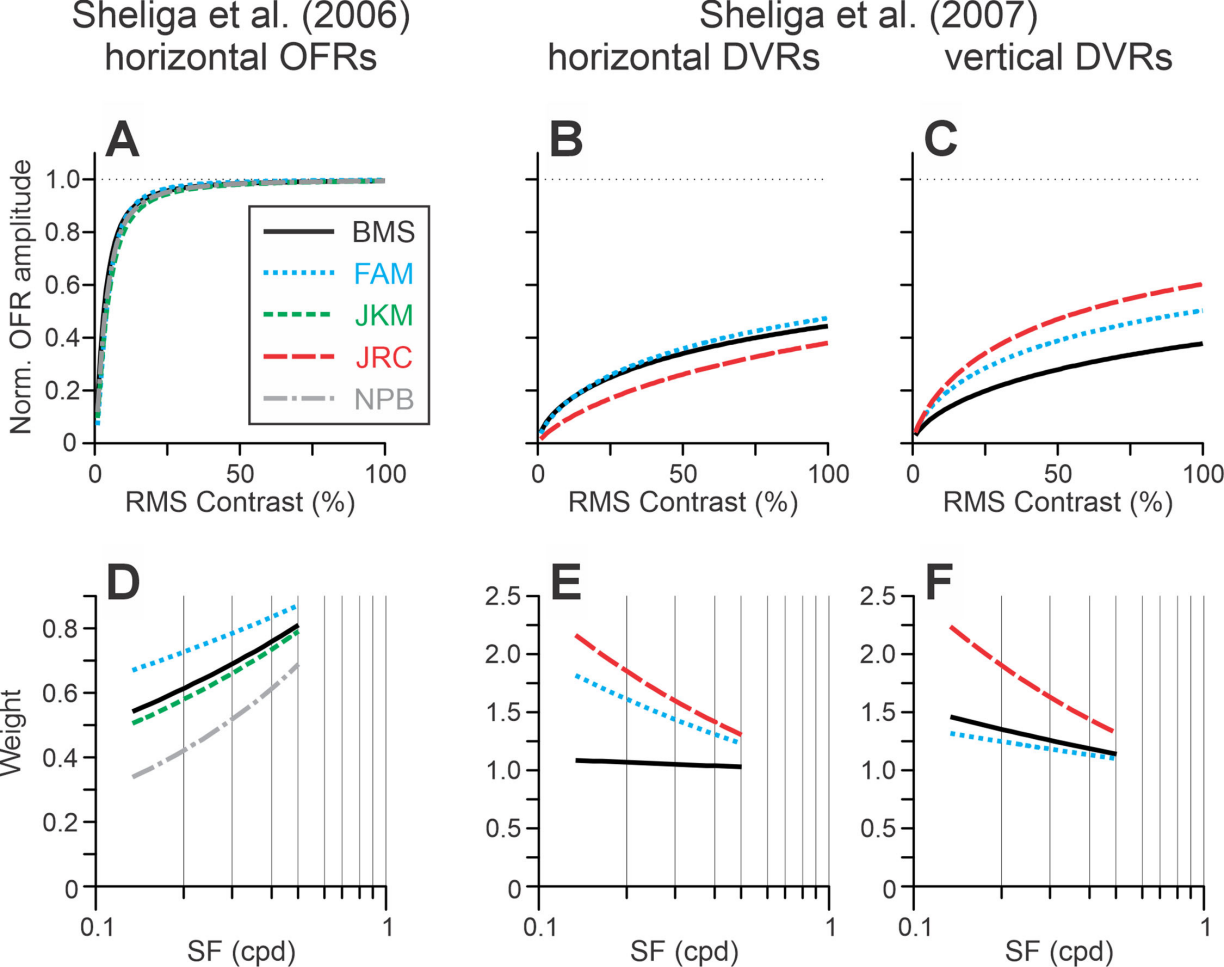
Model. The relationships between stimulus parameters and the model fits for each subject (color-coded; see rectangular insert) for horizontal OFRs in Sheliga et al. (2006b) (left column of panels) and horizontal (middle column of panels) and vertical DVRs (right column of panels) in Sheliga et al. (2007). (**A**–**C**) normalized Naka–Rushton equation (Equation 4c for OFRs and Equation 2 for DVRs). (**D**–**F**) The function relating *W* to SFs of sine-wave gratings (Equation 6). Table 2 lists the best-fit values of free parameters.

A competition between contributions of different Fourier components was modeled by a power-law summation, free parameter *m*. As Table 1 shows, however, the best-fit values of *m* were close to 1. Constraining *m* to equal 1 (i.e., assuming a linear summation) resulted in a statistically significant deterioration of fits in subject JC but not in subjects BMS or EJF: *F*(1, 56) = 7.38, *p* < 0.01; *F*(1, 86) = 3.35, *p* = 0.07; and *F*(1, 56) = 0.06, *p* = 0.80, respectively. A single Fourier component contribution (*k*), on the other hand, was highly nonlinear. Equating *k* to 1 led to a statistically significant deterioration of fits in all subjects (*p* < 0.002): *F*(1, 86) = 28.12, *F*(1, 56) = 25.24, and *F*(1, 56) = 10.91 for subjects BMS, EJF, and JC, respectively). In the Sheliga et al. (2022) study, the best fits to the data were achieved when the weights of components were modeled by a power function (figures 6C and 6D in Sheliga et al., 2022). Substituting Equation 4b by a power function led to a significant deterioration of fits in all subjects (*p* < 0.04). In the Sheliga et al. (2020) study, the best fits to the data were achieved when the weight function was the product of a power and an inverted cumulative log-Gaussian function (figure 7A in Sheliga et al., 2020). Making such a substitution did not result in statistically significant improvements of fits in any of the subjects (*p* > 0.22).

### Earlier studies: Sheliga et al. (2006b)

**Figure A1.**
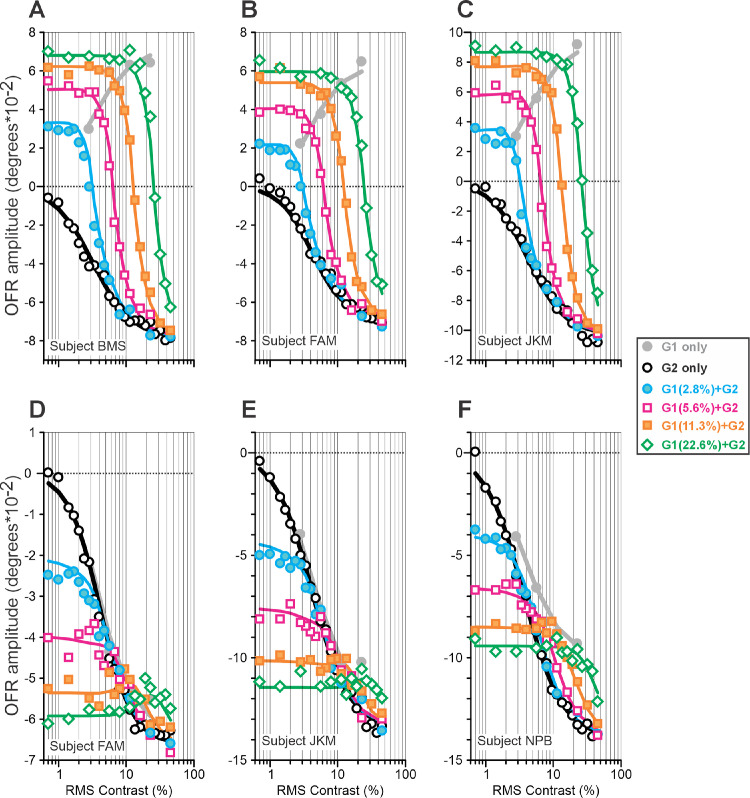
Data from Sheliga et al. (2006b). Data (symbols) and Equation 4 fits (lines). Upper row, 3f5f stimuli; lower row, 3f7f stimuli. Table 2 lists the best-fit values of free parameters. (**A**–**C**) Subjects BMS, FAM, and JKM, respectively. (**D**–**F**) Subjects FAM, JKM, and NPB, respectively.

Horizontal OFRs were recorded in this study in response to the horizontal motion of 1D vertical sine-wave gratings. Motion stimuli were either isolated gratings or a combination of two gratings. When combined, gratings either moved in the opposite direction and had SFs related as 3:5 (3f5f stimuli; Experiment 2) or moved in the same direction and had SFs related as 3:7 (3f7f stimuli; Experiment 3). The RMS contrast of one grating (G1) (Figure A1) was fixed at one of five levels (0%, 2.8%, 5.6%, 11.3%, or 22.6%), whereas the RMS contrast of the other (G2) (Figure A1) was fixed at one of 15 to 24 levels ranging from 0% to 45.4%. Two subjects participated in both experiments (FAM and JKM), two others in only one (BMS in Experiment 2 and NPB in Experiment 3).

The data were fit by Equation 4. Data of subjects FAM and JKM from Experiments 2 and 3 were fit by a single set of free parameters. The range of grating SFs used in these experiments was quite narrow: 0.20 and 0.33 cpd in Experiment 2, and 0.16 and 0.38 cpd in Experiment 3. We, therefore, did not use Equation 4a for calculating the contributions of component gratings; such a Gaussian function would have been poorly constrained. We, instead, fed *A_MAX(G_*_1_*_)_* and *A_MAX(G_*_2_*_)_* as free parameters to Equation 4: two free parameters for subjects BMS and NPB and four free parameters for subjects FAM and JKM. For the same reason (narrow range of SFs) we utilized a weight function, which was different from Equation 4b and had a single free parameter, *K_w_*:

(6) $$\begin{array}{c} W\left({\text{SF}}_{i}\right)={\text{SF}}_{i}^{K_{w}}\; \end{array}$$

In the Sheliga et al. (2020) OFR study, the best fits to the data were achieved when a function of weight was the product of a power and an inverted cumulative log-Gaussian function, and a limited range of low to intermediate SFs in the study by Sheliga et al. (2006b) should be readily accounted for by a power portion of it. In the Sheliga et al. (2022) DVR study, the best fits to the data were achieved when the weights of components were modeled by a power function for components of all SFs. Thus, the fits for subjects BMS and NPB had seven free parameters, whereas those for FAM and JKM had nine parameters.

Equation 4 provided very good fits to the data for all subjects (*r*^2^ = 0.993, 0.994, 0.995, and 0.990 for subjects BMS, FAM, JKM, and NPB, respectively). These fits are shown in Figure A1, and Table 2 lists the best-fit values of the free parameters. The left-side panels of Figure 6 present the best-fit normalized Naka–Rushton (Figure 6A; Equation 4c) and weight functions (Figure 6D; Equation 6). Constraining *m* to equal 1 (i.e., assuming a linear summation) resulted in a statistically significant deterioration of fits in all but one subject (NPB). Equating *k* to 1 led to statistically significant fit deterioration in all subjects (*p* < 10^–^^1^^2^). Setting equal weights for contributions of components in combined gratings led to fit deterioration in all subjects, as well (*p* < 0.002).

**Figure 7.**
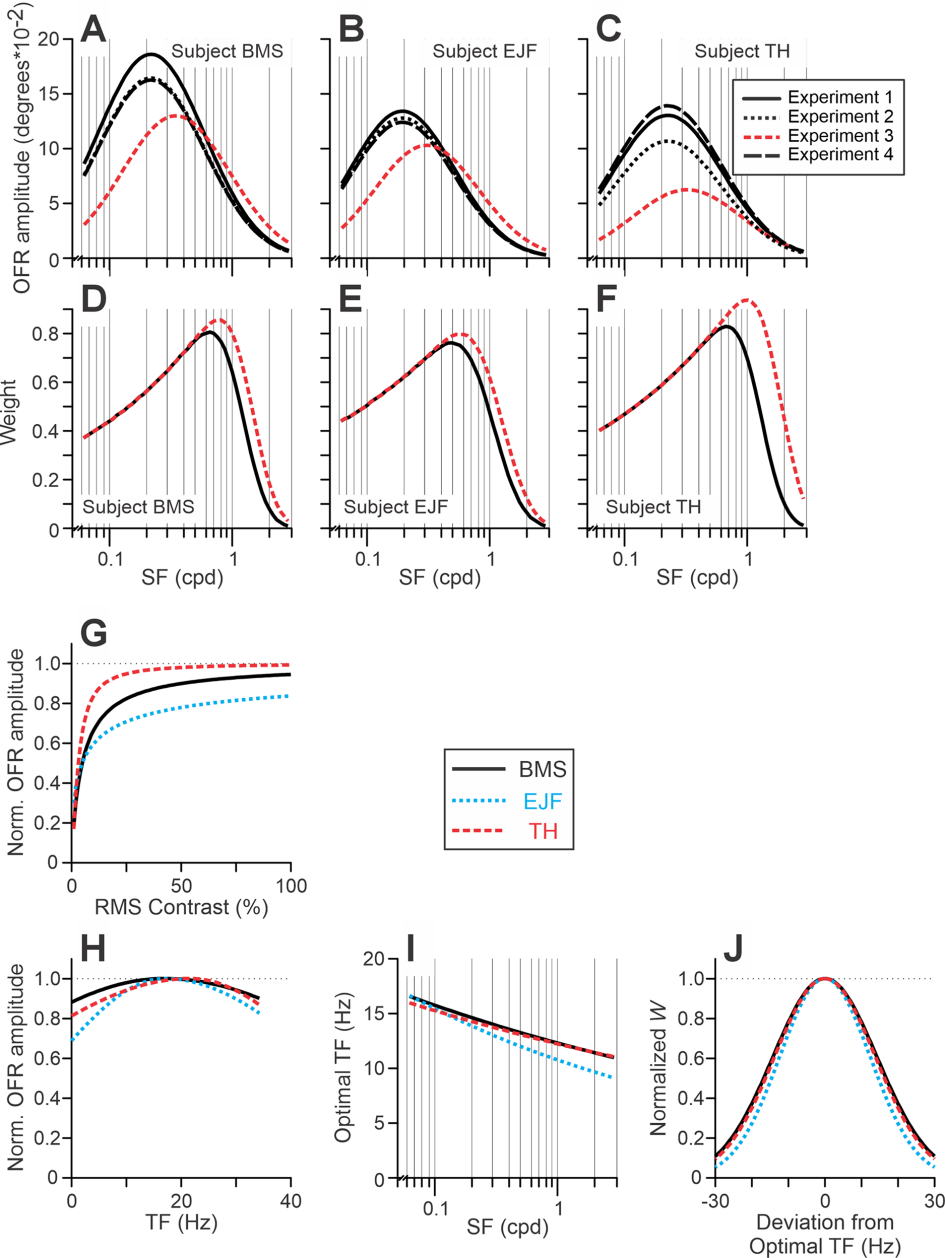
Model. The relationships between stimulus parameters and the model fits for each subject and experiment (color-coded; see rectangular inserts) in Sheliga et al. (2020). (**A**–**C**) Equation 4a, OFR SF tuning. (**D**–**F**) The product of Equations 4b and 6, the function relating SFs to *W*. (**G**) Equation 4c, the normalized Naka–Rushton equation. (**H**) OFR TF tuning. (**I**, **J**) A simple interaction between SFs and TF, where *W* is a Gaussian function of TF (J; Equation 7), but the TF at which *W* peaks (i.e., optimal TF) depends on the SF (I; Equation 8). Table 3 lists the best-fit values of free parameters.

**Table 2.** Best-fit parameters of Equation 4 (OFR data) and Equation 1 (DVR data) fits (Sheliga et al., 2006b; Sheliga et al., 2007).

Experiment	Subject	*C*_50_	*n_C_*	*k*	*m*	*K_W_*	*r* ^2^
OFR horizontal	BMS	0.03	1.50	3.2	1.5	0.30	0.993
	FAM	0.04	1.85	3.3	1.3	0.20	0.994
	JKM	0.04	1.57	3.9	1.2	0.34	0.995
	NPB	0.04	1.55	3.0	1.1	0.54	0.990
DVR horizontal	BMS	1.43	0.63	2.0	1.0	−0.04	0.994
	FAM	1.16	0.69	2.3	1.1	−0.30	0.998
	JRC	1.86	0.79	1.7	1.0	−0.38	0.991
DVR vertical	BMS	2.16	0.65	2.8	1.1	−0.19	0.998
	FAM	0.98	0.67	2.3	1.2	−0.14	0.999
	JRC	0.58	0.78	3.2	1.0	−0.40	0.996

### Earlier studies: Sheliga et al. (2007)

**Figure A2.**
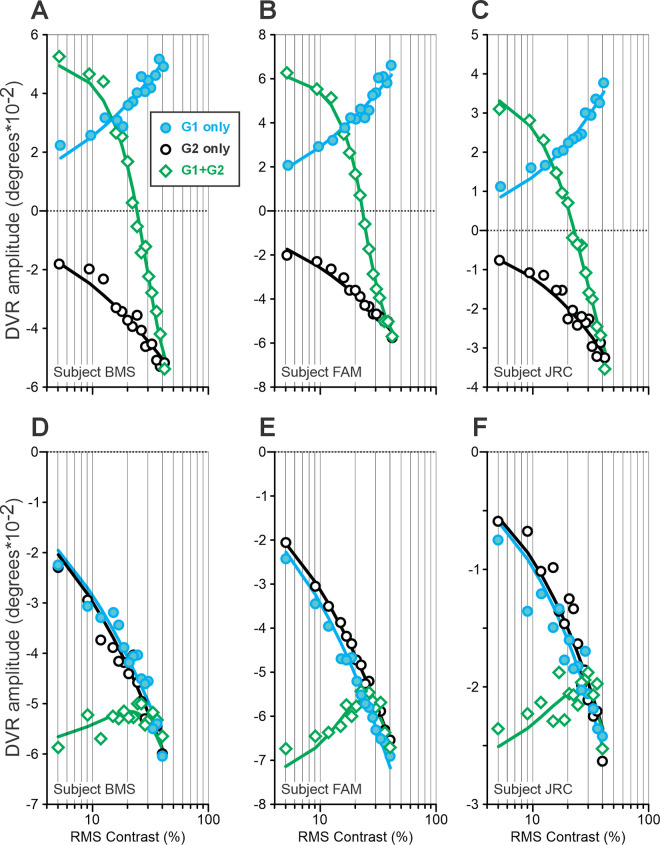
Horizontal DVR data from Sheliga et al. (2007). Data (symbols) and Equation 1 fits (lines). Upper row, 3f5f stimuli; lower row, 3f7f stimuli. Table 2 lists the best-fit values of free parameters.

**Figure A3.**
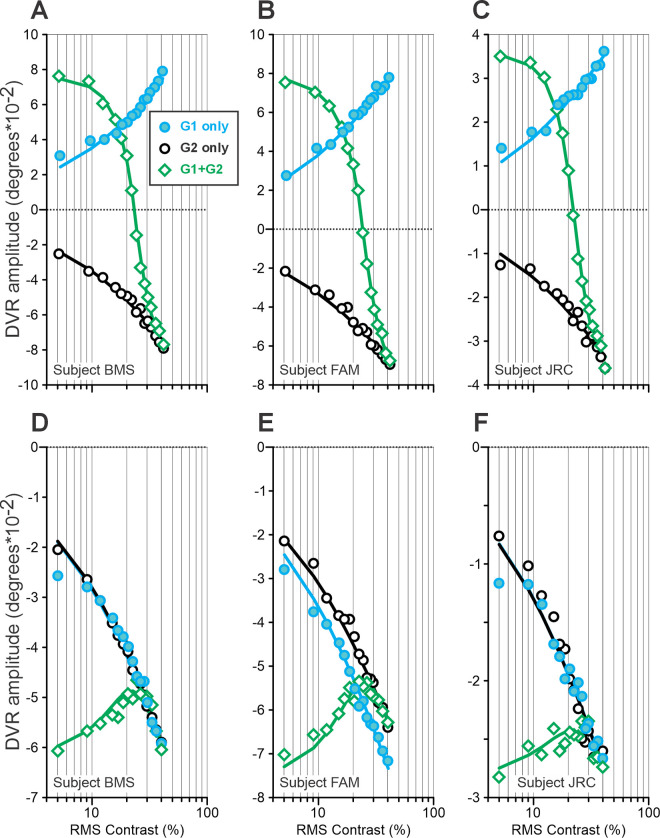
Vertical DVR data from Sheliga et al. (2007). Data (symbols) and Equation 1 fits (lines). Upper row, 3f5f stimuli; lower row, 3f7f stimuli. Table 2 lists the best-fit values of free parameters.

Horizontal and vertical DVRs were recorded in this study in response to the horizontal and vertical disparity of sine-wave gratings. Disparity stimuli were either isolated gratings or a combination of two gratings. When combined, two gratings either had disparities of opposite sign (crossed/uncrossed or left-hyper/right-hyper) and SFs related as 3:5 (3f5f stimuli; Experiments 1 and 2) or had disparities of the same sign and SFs related as 3:7 (3f7f stimuli; Experiment 3). The RMS contrast of a combined stimulus ranged from 31.7% to 42.4%, whereas the ratios of component grating contrasts were fixed at 0.125, 0.25, 0.3536, 0.5, 0.5946, 0.7071, 0.8409, 1.0, 1.1892, 1.4142, 1.6818, 2.0, 2.8284, 4.0, or 8.0. DVRs to isolated gratings with contrasts equal to the contrasts of each component in combined stimuli were also recorded (G1 and G2 in Figures A2 and A3). Three subjects participated in all experiments (BMS, FAM, and JRC).

The data were fit by Equation 1. The 3f5f and 3f7f data of either disparity were fit by a single set of free parameters, and the sets were different for horizontal and vertical DVRs. The range of grating SFs used in these experiments was narrow—from 0.11 to 0.7 cpd—so we did not use Equation 4a but instead fed *A_MAX(G_*_1_*_)_* and *A_MAX(G_*_2_*_)_* as free parameters to Equation 1, with four free parameters per set. As in the Sheliga et al. (2022) DVR study, we utilized Equation 6 to model weights of the components; thus, the fits had nine free parameters.

Equation 1 provided very good fits to the data for all subjects (median *r*^2^ = 0.997; range, 0.991–0.999). The fits are shown in Figure A2 (horizontal DVRs) and Figure A3 (vertical DVRs). Table 2 lists the best-fit values of the free parameters. The middle (horizontal DVRs) and the right-side (vertical DVRs) panels of Figure 6 present the best-fit normalized Naka–Rushton (Figures 6B and 6C; Equation 4c) and weight functions (Figures 6E and 6F; Equation 6). The best-fit *m* values were close to 1. Equating *m* to 1 (i.e., assuming a linear summation) resulted in a statistically significant deterioration of fits in subject FAM and for vertical DVRs in subject BMS. Equating *k* to 1 led to statistically significant fit deterioration in all subjects (*p* < 10^–^^7^). Setting weights for contributions of components to be equal in combined gratings resulted in statistically significant fit deterioration in all but one subject (BMS; horizontal DVRs). Fitting horizontal and vertical DVR data by a single set of free parameters resulted in a statistically significant fit deterioration in all subjects (*p* < 10^–6^).

### Earlier studies: Sheliga et al. (2020)

Horizontal OFRs were recorded in response to the horizontal motion of 1D vertical sine-wave gratings. Motion stimuli were either isolated gratings or a combination of two gratings (Experiments 1–4) or three gratings (Experiment 4). In Experiments 1 to 3, when combined, gratings moved in the opposite directions. In Experiment 4, in two-grating stimuli, gratings could move in either the same or opposite directions. In the three-grating stimuli, two moved in the same direction and the direction of the third one was opposite to that. SFs of gratings ranged from 0.04 to 1.3 cpd. The RMS contrast of one grating (G1; Figures A5 to A8) was always fixed at 8.5% (as was the contrast of G3 in Experiment 4), whereas the RMS contrast of the other (G2; Figures A5 to A8) could have one of three levels: 4.9%, 8.5%, or 14.7%. Motion TF was fixed at 18-3/4 Hz in Experiments 1, 3, and 4. It varied in Experiment 2: 3-1/8, 6-1/4, 12-1/2, 18-3/4, 25, or 30 Hz. The sizes of the visual stimuli in Experiments 1, 2, and 4 were ∼22° × ∼22° (512 × 512 pixels); in Experiment 3, ∼11° × ∼11° (256 × 256 pixels). Three subjects participated in all experiments (BMS, EJF, and TH).

The data of Experiments 1 to 4 were fit by Equation 4 using a single set of free parameters, except for µ*_SF_*, µ*_W_*, and *A_MAX_*. Following the modeling results of Sheliga et al. (2020) and the current paper, µ*_SF_* and µ*_W_* were allowed to be different in Experiment 3, in which the area of visual stimuli was 1/4 of that in Experiments 1, 2, and 4. A separate free parameter was allowed for *A_MAX_* in each experiment: Experiments 1 to 4 were run in separate sessions lacking a common stimulus condition, which would permit a between-experiment response normalization. OFR TF tuning was modeled by an asymmetric Gaussian function, allowing different σ values (σ1*_TF_* and σ2*_TF_*) at TFs lower and higher than the Gaussian offset (µ*_TF_*), respectively. The amplitude of this Gaussian was set to 1. Using sine waves, Sheliga et al. (2016) showed that the OFR TF tuning did not depend on stimulus SFs. Thus, the response to a Fourier component (*OFR_i_*) was calculated as the product of the value produced by Equation 4a (the dependence upon the SF of this component) and the value produced by the TF tuning function (the dependence upon the motion TF of this component). In the Sheliga et al. (2020) study, the best fits were achieved when the dependence of the weight function on the SF of a component grating was the product of a power (Equation 6) and an inverted cumulative log-Gaussian function (Equation 4b). Sheliga et al. (2020) also showed that the weights depended on motion TF; the weights were described by an interaction between SF and TF, where the weight was a Gaussian function of TF, but the TF at which the weight peaks depended on SF:

(7) $$\begin{array}{c} W\left({\text{SF}}_{i};{\text{TF}}_{i}\right)=e^{-\frac{{\left[g\left({\text{SF}}_{i}\right)-{\text{TF}}_{i}\right]}^{2}}{2*\sigma_{TFw}^{2}}}\; \end{array}$$

where

(8) $$\begin{array}{c} g\left({\text{SF}}_{i}\right)=A_{TFw}*SF_{i}^{K_{TFw}}\; \end{array}$$

We utilized all of these earlier findings in formulating a weight function in the model. Thus, in total, the model had 21 free parameters.

**Figure A4.**
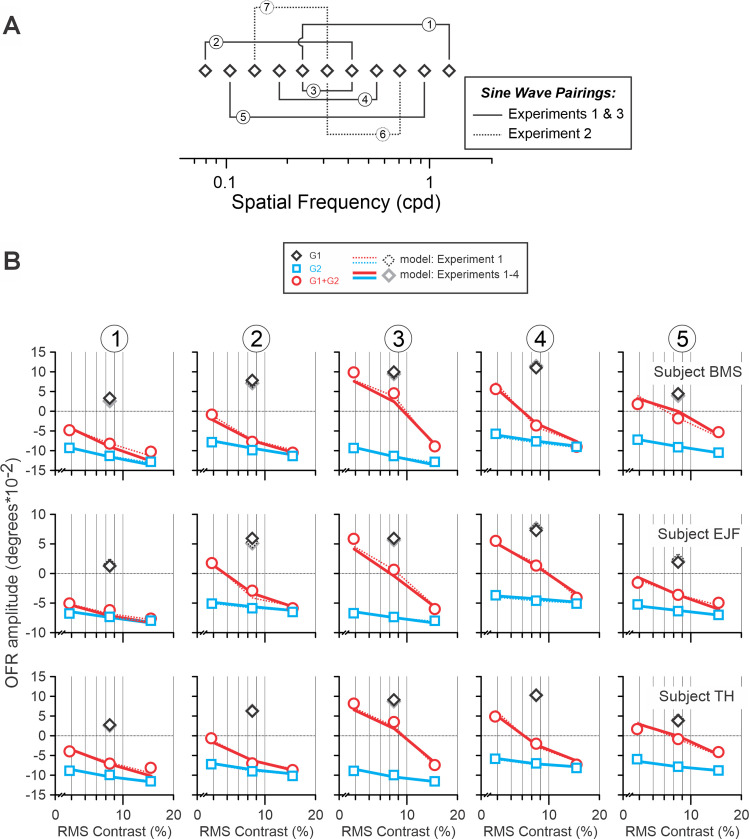
Data from Sheliga et al. (2020). (**A**) SFs of sine-wave gratings used in Experiments 1, 2, and 3, shown as diamond symbols arranged along the spatial frequency axis. Solid thin lines (numbered from 1 to 5) connect SFs that form two-component stimuli of Experiments 1 and 3; dotted thin lines (numbered 6 and 7) connect SFs forming two-component stimuli of Experiment 2. (**B**) Experiment 1. Data (symbols) and Equation 4 fits (lines). The data for each subject occupy a single row of panels. Different columns of panels show the data for different combinations of sine-wave gratings (indicated in panel **A**). Table 3 lists the best-fit values of free parameters.

**Figure A5.**
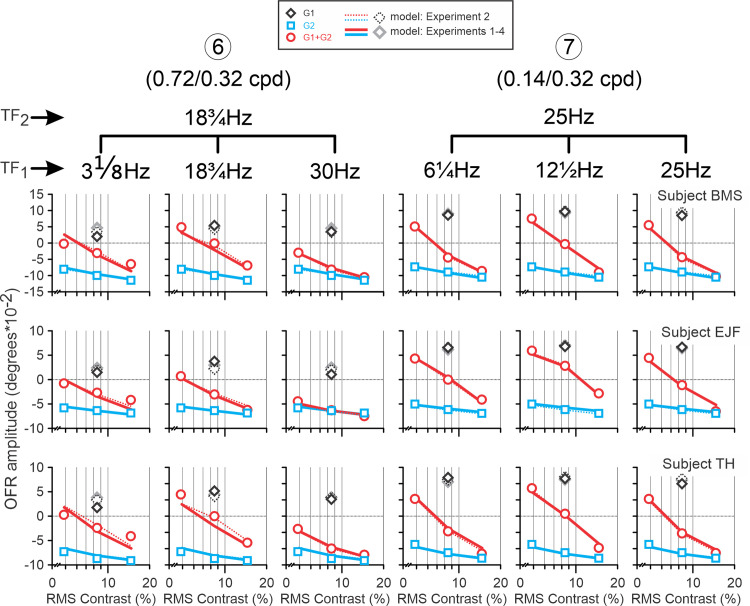
Data from Sheliga et al. (2020). Experiment 2. Data (symbols) and Equation 4 fits (symbols and lines; see rectangular insert). The data for each subject occupy a single row of panels. Different columns of panels show the data for different combinations of sine-wave gratings (indicated in Figure A4a). Table 3 lists the best-fit values of free parameters.

**Figure A6.**
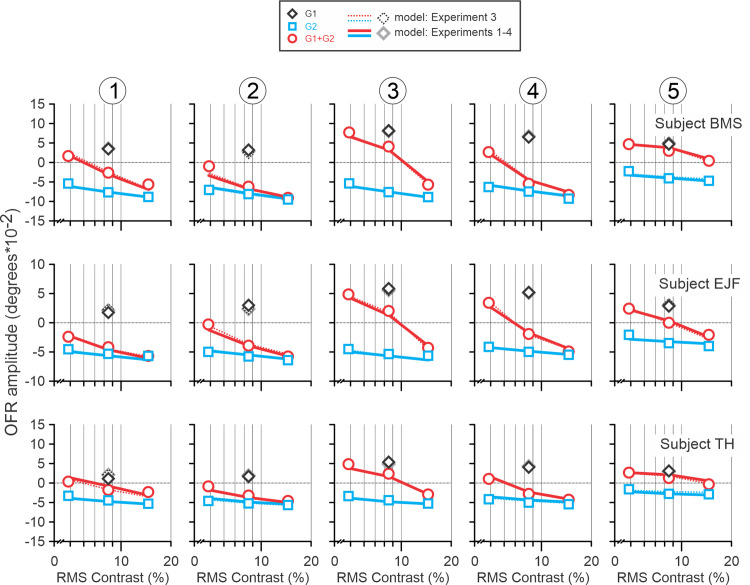
Data from Sheliga et al. (2020). Experiment 3. Data (symbols) and Equation 4 fits (symbols and lines; see rectangular insert). The data for each subject occupy a single row of panels. Different columns of panels show the data for different combinations of sine-wave gratings (indicated in Figure A4A). Table 3 lists the best-fit values of free parameters.

**Figure A7.**
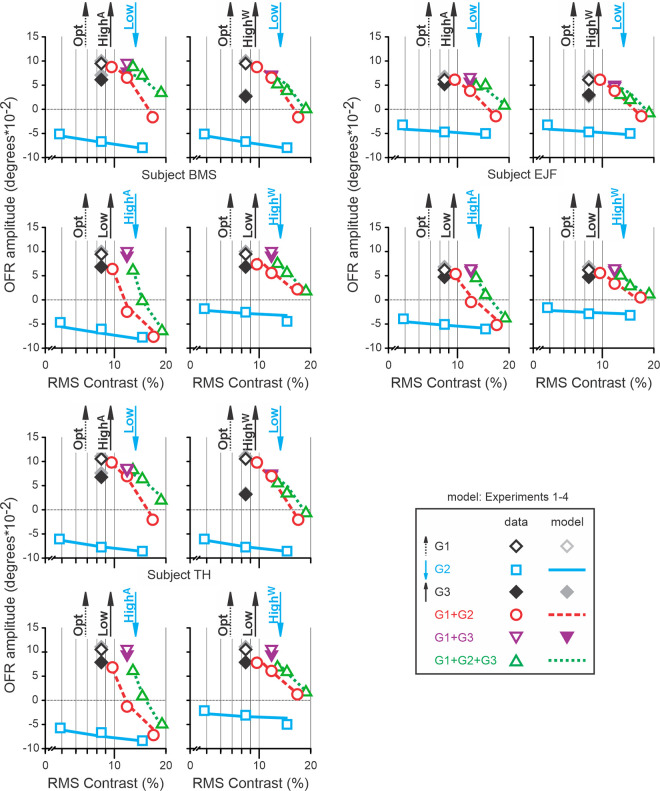
Data from Sheliga et al. (2020). Experiment 4. Data (symbols) and Equation 4 fits (symbols and lines; see rectangular insert). The data for each subject occupy a four-panel cluster. Table 3 lists the best-fit values of free parameters.

**Figure A8.**
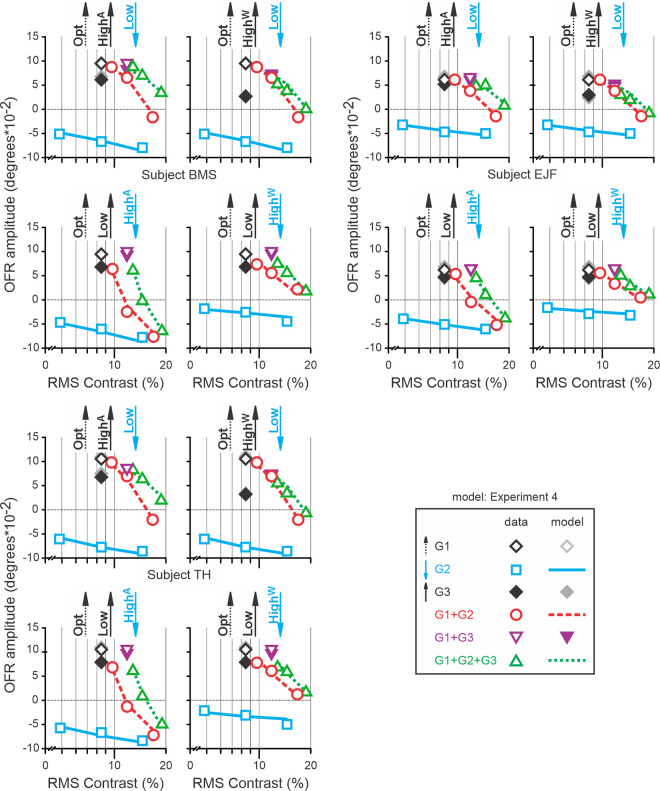
Data from Sheliga et al. (2020). Experiment 4. Data (symbols) and Equation 4 fits when the data of Experiment 4 were fit by a separate set of free parameters (symbols and lines; see rectangular insert). The data for each subject occupy a four-panel cluster. Table 3 lists the best-fit values of free parameters.

The resulting fits were good (*r*^2^ = 0.983, 0.985, and 0.984 for subjects BMS, EJF, and TH, respectively). The fits are shown in Figures A4 to A7 by thick solid lines. Table 3 lists the best-fit values of free parameters. Different panels of Figure 7 present the best-fit OFR SF tuning functions (Figures 7A to 7C; Equation 4a), weight function dependences on SF (Figures 7D to 7F; the product of Equations 4b and 6), normalized Naka–Rushton function (Figure 7G; Equation 4c), OFR TF tuning functions (Figure 7H), and the dependences of weight functions on an interaction between SF and TF: the optimal TF dependence on SF (Figure 7I; Equation 8) and the weight as a function of TF deviation from the optimal value (Figure 7J; Equation 7). A reduction in the number of free parameters from 64 (16 free parameters × 4 experiments) to 21 did not result in a statistically significant deterioration of fits: *F*(43, 62) = 0.73, *F*(43, 62) = 0.64, and *F*(43, 62) = 0.51 for subjects BMS, EJF, and TH, respectively. The 16 free-parameter fits for each experiment are shown by thin dotted lines in Figures A4 to A6 and by thick solid lines in Figure A8. Equating *m* to 1 (i.e., assuming a linear summation) resulted in a deterioration of fits in all subjects (*p* < 0.001), as did equating *k* to 1 (*p* < 10^–21^). Dropping the power portion from the dependence of weights on SF (i.e., using solely Equation 4b) resulted in fit deterioration in all subjects, as well (*p* < 10^–^^1^^2^). Removing the dependence of weights on motion TF also led to fit deterioration in all subjects (*p* < 10^–^^11^).

**Table 3.** Best-fit parameters of Equation 4 fits (Sheliga et al., 2020).

Subject	Experiment	*A_MAX_* (°)	µ*_SF_* (cpd)	σ*_SF_* (log_2_ units)	*C*_50_	*n_C_*	*k*	*m*	*K_W_*	µ*_W_* (cpd)	σ*_W_* (log_2_ units)	µ*_TF_* (Hz)	σ_1_*_TF_* (Hz)	σ_2_*_TF_* (Hz)	*A_TFw_* (Hz)	*K_TFw_*	σ*_TFw_* (Hz)	*r* ^2^
BMS	1	0.19	0.22	1.44	0.05	0.94	2.4	1.4	0.36	1.14	0.52	14.8	29.4	33.1	12.3	−0.11	14.2	0.983
	2	0.16																
	4	0.16																
	3	0.13	0.34							1.36								
EJF	1	0.13	0.19	1.41	0.05	0.54	2.4	1.3	0.30	0.97	0.63	14.9	17.2	24.5	10.8	−0.16	12.4	0.985
	2	0.13																
	4	0.12																
	3	0.10	0.30							1.14								
TH	1	0.13	0.22	1.46	0.03	1.39	2.1	1.4	0.33	1.19	0.52	19.0	29.5	20.6	12.2	−0.10	13.8	0.984
	2	0.11																
	4	0.14																
	3	0.06	0.32							1.73								

### Earlier studies: Quaia et al. (2017b)

Vertical OFRs were recorded in response to the vertical motion of 1D horizontal sine-wave gratings. Visual stimuli were either isolated moving gratings or a combination of two gratings: moving (stimulus) and stationary (mask). In the first experiment, the RMS contrasts of the mask and the stimulus were fixed at 22.6%. The SFs of stimuli were 0.125, 0.25, or 0.5 cpd, whereas those of the masks could vary from 0.0625 to 2 cpd in octave increments. In the second experiment, the SFs of the stimuli and masks were fixed at 0.25 and 0.5 cpd, respectively, whereas their RMS contrasts could vary from 1.8% to 28.2% in octave increments. In the third experiment, the range of stimulus/mask contrasts was the same as in the second experiment, whereas the SFs of stimuli and masks were fixed at 0.125 and 1 cpd, respectively. Three subjects participated in the first and second experiments (S1–S3), whereas only two subjects participated in the third (S1 and S3). Quaia et al. (2017b) used normalized OFRs when plotting the data in their figure 2 (i.e., the ratio between the OFR measured when the mask is present and that induced by the moving grating alone). To test our model, we used the Quaia et al. (2017b) raw data—the average velocity during a 70-ms open-loop interval—with the OFRs to isolated moving gratings also included, which were kindly provided to us by Christian Quaia, PhD.

The data were fit by the following equation:

(9) $$\begin{array}{c} \text{OFR}=\frac{C^{n}}{C^{n}+C_{50}^{n}}*\frac{{\text{OFR}}_{S}*{\left(W_{S}*C_{S}\right)}^{k}}{{\left(W_{S}*C_{S}\right)}^{k}+{\left(W_{M}*C_{M}\right)}^{k}}\; \end{array}$$

Equation 9 is a simplified version of Equation 4—namely, only the stimulus drives the OFRs, whereas both the stimulus and the mask contribute to the contrast normalization of the responses. The data of three experiments (two with S2) were fit by a single set of free parameters, except for *A_MAX_*. Experiments were run in separate sessions without a common stimulus condition; hence, there was no between-experiment response normalization. The dependence of weight function on SF was modeled as the product of Equations 6 and 4b and was the same for the stimulus and the mask. In total, the model had 11 (S1 and S3) or 10 (S2) free parameters.

**Figure 8.**
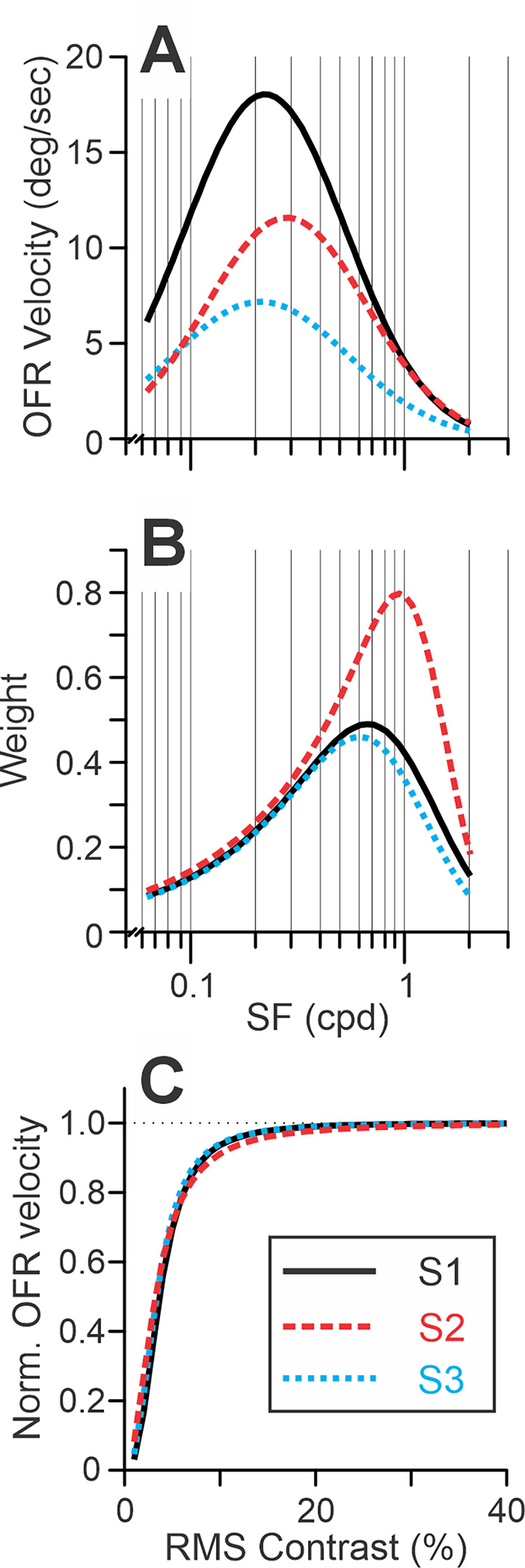
Model. The relationships between stimulus parameters and the model fits for each subject (color-coded; see rectangular insert) in Quaia et al. (2017b). (**A**) Equation 4a, OFR SF tuning. (**B**) The product of Equations 4b and 6, the function relating SFs to *W*. (**C**) Equation 4c, normalized Naka–Rushton equation. Table 4 lists the best-fit values of free parameters.

**Figure A9.**
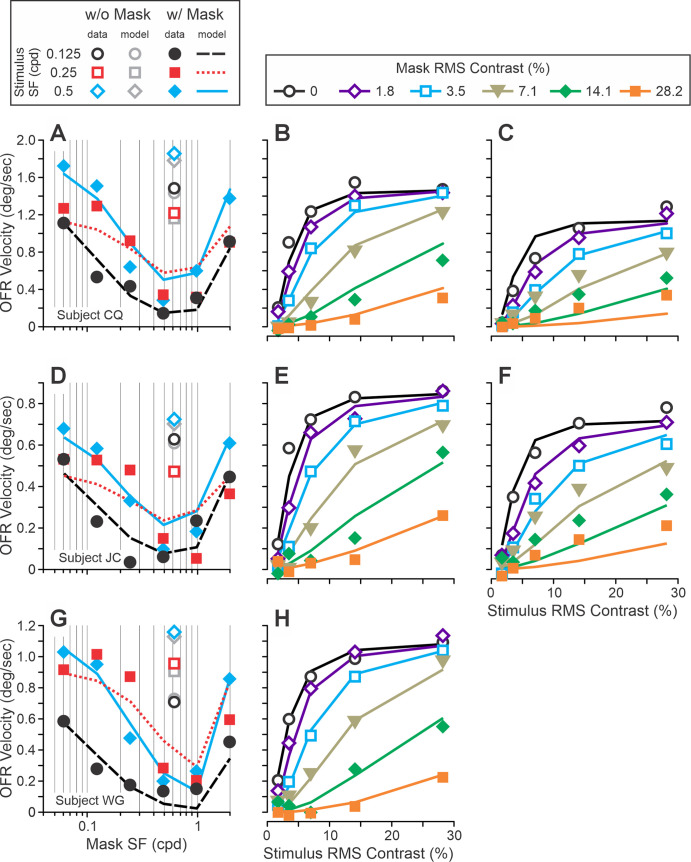
Data from Quaia et al. (2017b). Data (symbols) and Equation 4 fits (symbols and lines; see rectangular insert). The data for each subject occupy a single row of panels. Left, middle, and right columns of panels show the data for the first, second, and third experiments, respectively. Table 4 lists the best-fit values of free parameters.

The resulting fits were good (*r*^2^ = 0.954, 0.963, and 0.941 for subjects S1–S3, respectively). The fits are shown in Figure A9. Table 4 lists the best-fit values of free parameters. Figure 8A presents the best-fit OFR SF tuning functions (Equation 4a); Figure 8B, the weight functions (the product of Equations 4b and 6); and Figure 8C, the normalized Naka–Rushton function (Equation 4c). Equating *k* to 1 led to fit deterioration in all subjects (*p* < 10^–6^). Dropping the power portion from the dependence of weight on SF (i.e., using solely Equation 4b) resulted in fit deterioration in all subjects, as well (*p* < 10^–17^). In Equation 9, omitting the weight scaling free parameter for the stimulus is justified, because *W_S_* appears in both the numerator and the denominator; hence, a scaling parameter gets canceled out. This is not the case for the *W_M_*, which is present only in the denominator. Allowing an additional free parameter for a *W_M_* scaling coefficient led to statistically significant improvements in fits for subject S1 (coefficient 1.20; *p* < 0.005), but not for the other two subjects (1.06, *p* > 0.35 for subject S2 and 1.09, *p* > 0.25 for subject S3).

**Table 4.** Best-fit parameters of Equation 4 fits (Quaia et al., 2017b).

Subject	Figure No. in Quaia et al. (2017b)	*A_MAX_* (°/s)	µ*_SF_* (cpd)	σ*_SF_* (log_2_ units)	*C*_50_	*n_C_*	*k*	*K_W_*	µ*_W_* (cpd)	σ*_W_* (log_2_ units)	*r* ^2^
S1	2	1.80	0.22	1.25	0.04	2.66	2.0	0.89	0.89	0.79	0.954
	3	1.48									
	4	1.43									
S2	2	1.16	0.28	1.23	0.03	2.06	2.2	0.84	1.31	0.48	0.963
	3	1.10									
S3	2	0.72	0.21	1.36	0.03	2.51	1.8	0.90	0.82	0.75	0.941
	3	0.86									
	4	0.84									

The Quaia et al. (2017b) study is of particular interest. The authors combined moving sine-wave gratings with stationary ones. However, our model was able to account for these data, as well: stationary gratings did not contribute to motion drive but their presence in the stimulus contributed to global contrast normalization.

### Earlier studies: Sheliga et al. (2016)

Horizontal OFRs were recorded in response to the translational horizontal motion of 1D vertical unfiltered and filtered (bandpass and notch Gaussian filters on a log scale) white noise stimuli. In all experiments, the size of visual stimuli was ∼25° × ∼25° (512 × 512 pixels). In Experiment 1, unfiltered and notch-filtered stimuli moved at 22°/s, 44°/s, or 88°/s; bandpass-filtered stimuli were also included but moved at 44°/s only. The central SF of the filter varied from 0.0625 to 4 cpd in half-octave increments, whereas the FWHM was 2 octaves. In Experiment 1B, vertical sine-wave gratings shifted horizontally with a range of TFs (1.67, 2.5, 4.17, 6.25, 8.33, 12.5, 16.7, 25, and 37.5 Hz), and two SFs were tested in each subject: 0.125 and 0.5 cpd. In Experiment 1C, only bandpass-filtered white noise stimuli were used (central SFs varied from 0.0625 to 2 cpd in octave increments; 2-octave FWHM), with the speed of each stimulus chosen such that its central SF moved with the near-optimal TF for each subject. In Experiment 2, white noise stimuli were lowpass filtered by removing Fourier components whose SFs were greater than 0.71 cpd. Stimuli were then passed through notch filters as in Experiment 1. In Experiment 3, unfiltered white noise stimuli moved horizontally at a range of different speeds (15°/s, 22°/s, 29°/s, 36°/s, 51°/s, 73°/s, or 109°/s). Three subjects participated in all experiments (AGB, BMS, and EJF).

Our current study also uses unfiltered, bandpass, and notch-filtered white noise stimuli, but Sheliga et al. (2016) studied translational motion and thus there was no change in the frame-to-frame appearance of the stimulus (it just shifted horizontally), whereas in the current study the phases of all Fourier components shifted each video frame by 1/8th of their respective wavelengths in the same direction, leading to changes in the frame-to-frame appearance of the stimulus; compare the two panels of Figure 1E.

The data of Experiments 1, 1B, 1C, 2, and 3 were fit by Equation 4 using a single set of free parameters, except for *A_MAX_*. The fits for 44°/s stimuli in Experiment 1, for sine-wave gratings in Experiment 1B, and for bandpass-filtered stimuli in Experiment 1C were allowed to have a separate free parameter for *A_MAX_*, because they were run in separate sessions that lacked a common stimulus condition, precluding a between-experiment response normalization. OFR TF tuning was modeled by an asymmetric Gaussian function, allowing different σ values (σ1*_TF_* and σ2*_TF_*) at TFs lower and higher than the Gaussian offset (µ*_TF_*), respectively. The amplitude of this Gaussian was set to 1. Thus, the response to a Fourier component (*OFR_i_*) was calculated as the product of the value produced by Equation 4a (the dependence upon the SF of this component) and the value produced by the TF tuning function (the dependence upon the motion TF of this component). The weight function dependence on the Fourier component SFs was modeled by an inverted cumulative log-Gaussian function (Equation 4b). In addition, σ*_SF_* and σ*_W_* were constrained to be the same, as was done in the analyses of the current paper. Thus, in total, the model had 14 free parameters.

**Figure 9.**
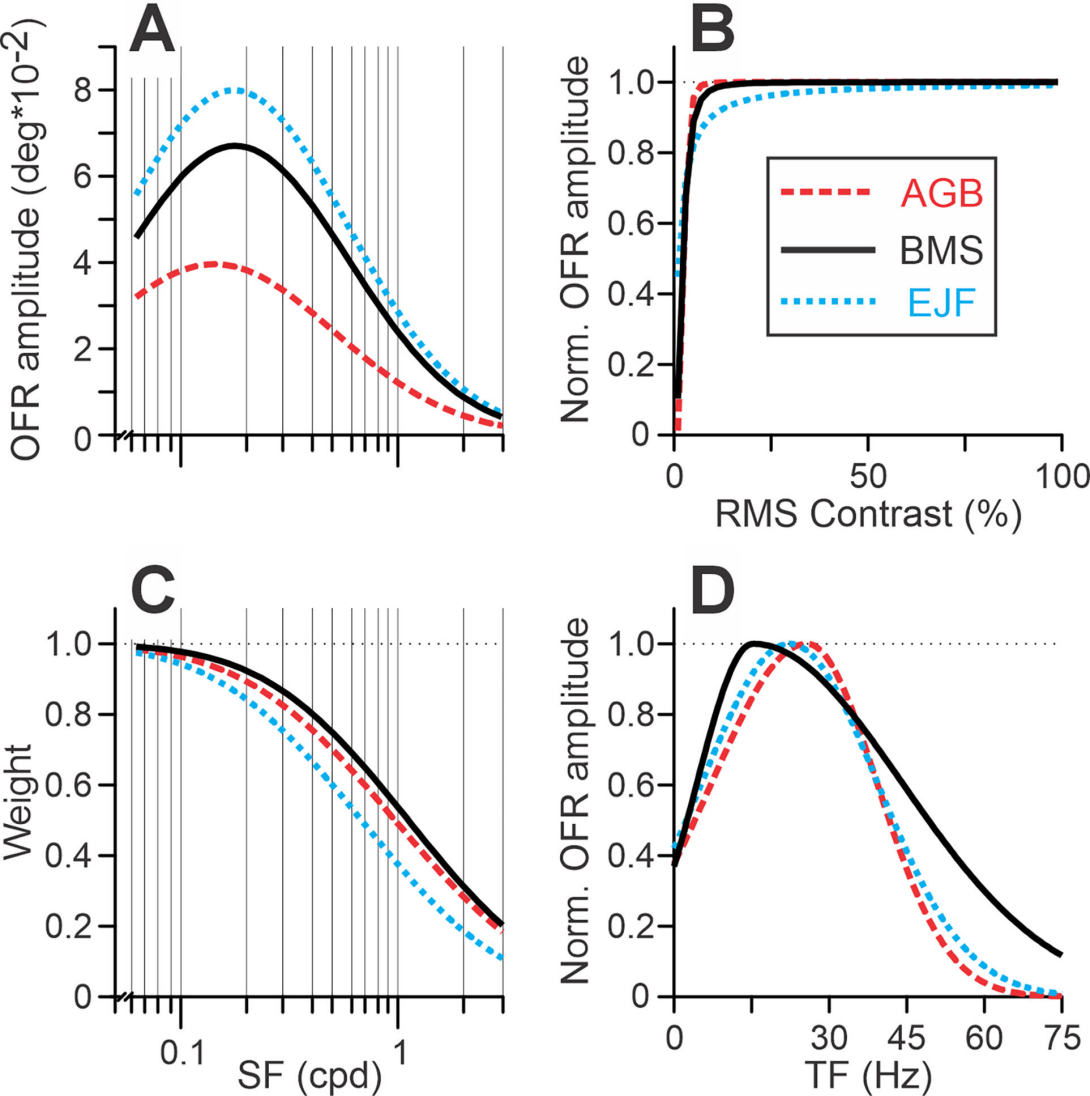
Model. The relationships between stimulus parameters and the model fits for each subject (color-coded; see rectangular insert) in Sheliga et al. (2016). (**A**) Equation 4a, OFR SF tuning. (**B**) Equation 4c, normalized Naka–Rushton equation. (**C**) Equation 4b, the function relating SFs of the Fourier components to *W*. (**D**) OFR TF tuning. Table 5 lists the best-fit values of free parameters.

**Figure A10.**
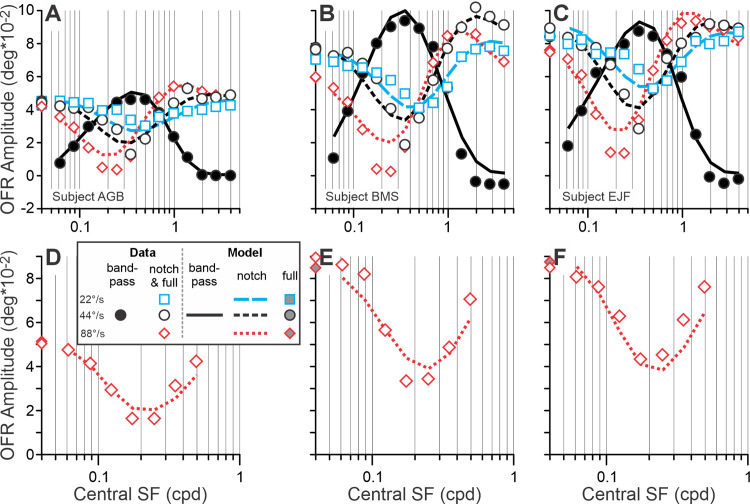
Data from Sheliga et al. (2016). Data (symbols) and Equation 4 fits (symbols and lines; see rectangular insert). The data for each subject occupy a single column. Upper row, Experiment 1; lower row, Experiment 2. Table 5 lists the best-fit values of free parameters.

**Figure A11.**
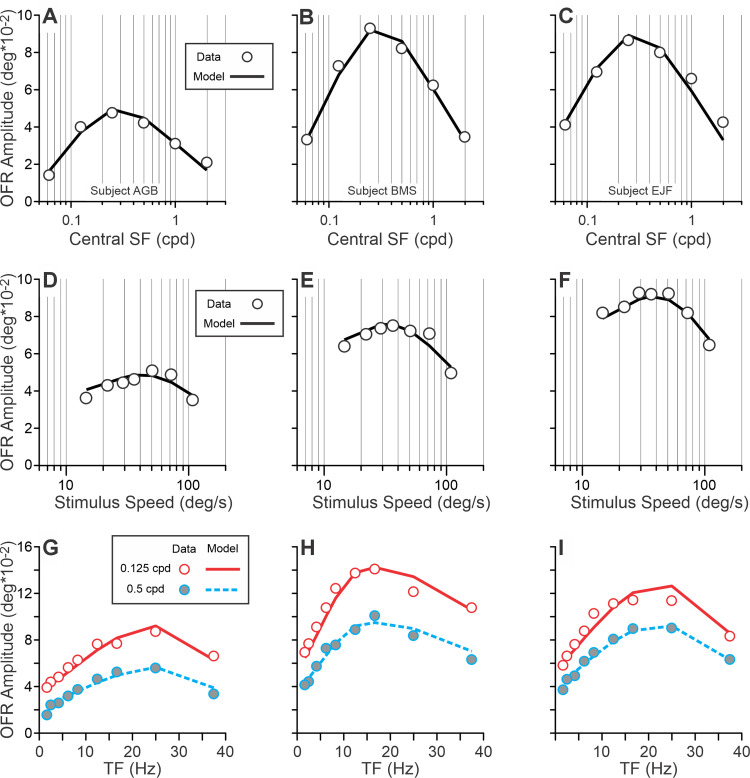
Data from Sheliga et al. (2016). Data (symbols) and Equation 4 fits (lines). See rectangular inserts. The data for each subject occupy a single column. Upper row, Experiment 1C; middle row, Experiment 3; lower row, Experiment 1B. Table 5 lists the best-fit values of free parameters.

The fits were quite good (*r*^2^ = 0.946, 0.954, and 0.937 for subjects AGB, BMS, and EJF, respectively). They are shown in Figures A10 and A11. Table 5 lists the best-fit values of free parameters. Figure 9 presents the best-fit OFR SF tuning functions (Figure 9A; Equation 4a), normalized Naka–Rushton function (Figure 9B; Equation 4c), weight dependences on SF (Figure 9C; Equation 4b), and the OFR TF tuning functions (Figure 9D). Equating *k* or *m* to 1 led to fit deterioration in all subjects (*p* < 0.001). Adding the dependence of the weight function on the motion TFs of the components (three additional free parameters; Equations 7 and 8) did not improve the fits. However, removing the weight function from Equation 4 altogether led to a statistically significant fit deterioration in all subjects (*p* < 10^–^^2^^8^). Allowing σ*_SF_* and σ*_W_* to be different did not improve the fits in subjects AGB and BMS, although for EJF the improvement was statistically significant (*p* < 0.001; 1.89 and 1.40 as the best-fit values for σ*_SF_* and σ*_W_*, respectively).

**Table 5.** Best-fit parameters of Equation 4 fits (Sheliga et al., 2016).

Subject	Experiment	*A_MAX_* (°)	µ*_SF_* (cpd)	σ*_SF_* and σ*_W_* (log_2_ units)	*C*_50_	*n_C_*	*k*	*m*	µ*_TF_* (Hz)	σ1*_TF_* (Hz)	σ2*_TF_* (Hz)	µ*_W_* (cpd)	*r* ^2^
AGB	1 (22°/s, 88°/s), 2, and 3	0.040	0.14	1.82	0.03	4.6	3.3	0.75	25.7	18.4	13.5	0.96	0.946
	1 (44°/s)	0.036											
	1B	0.093											
	1C	0.034											
BMS	1 (22°/s, 88°/s), 2, and 3	0.067	0.18	1.72	0.02	2.6	2.4	0.79	15.3	10.8	28.8	1.10	0.954
	1 (44°/s)	0.070											
	1B	0.147											
	1C	0.065											
EJF	1 (22°/s, 88°/s), 2, and 3	0.080	0.17	1.74	0.01	1.1	2.3	0.78	22.3	17.0	17.0	0.67	0.937
	1 (44°/s)	0.074											
	1B	0.417											
	1C	0.070											

## Discussion

In this paper, we show that the model proposed earlier to account for the DVRs in response to horizontal and vertical disparity steps of white noise visual stimuli (Sheliga et al., 2022) provides an excellent description of the short-latency ocular responses in the visual motion domain, as well. We also reanalyzed the data and applied the model to several earlier OFR and DVR studies that used sine-wave gratings (single or a combination of two or three gratings) and white noise stimuli. The model provided a very good account of all of these data. The model posits that the short-latency eye movements—OFRs and DVRs—can be accounted for by the operation of two factors: an excitatory drive, determined by a weighted sum of contributions of stimulus Fourier components, scaled by a global contrast normalization mechanism. The output of the operation of these two factors is then nonlinearly scaled by the total contrast of the stimulus. The contrast normalization is believed to reflect the divisive inhibition among populations of cortical neurons sensitive to different SF components of visual stimuli (Britten & Heuer, 1999; Carandini & Heeger, 1994; Carandini, Heeger, & Movshon, 1997; Heuer & Britten, 2002; Simoncelli & Heeger, 1998). A power law summation mechanism was also successfully applied to account for spatial summation properties of neurons in cortical motion area MT (e.g., Britten & Heuer, 1999). On a grand scale, it is quite noteworthy that, despite the different roles of disparity (horizontal and vertical) and motion signals in visual scene analyses, the earliest processing stages of these different signals appear to be very similar.

Several parameters of the model, however, do end up being very different for various types of visual stimuli. On one hand, fitting the responses to disparity versus motion stimuli requires the use of quite dissimilar SF-dependent weight functions. The idea that the contributions of different components are weighted is not new (e.g., Meso, Gekas, Mamassian, & Masson, 2022; Miura, Inaba, Aoki, & Kawano, 2014a). For disparity stimuli, the weights are a power function of SF (Equation 6) with a negative power coefficient; that is, the weights of the components become smaller as the component SF goes up (Figures 6E and 6F; see also figures 6C and 6D in Sheliga et al., 2022). For motion stimuli, the weights are an inverted cumulative log-Gaussian function (for broadband stimuli) or the product of a power function and an inverted cumulative log-Gaussian function (for two or three combined sine-wave gratings). The coefficient of the power function, however, is positive; that is, the weights of the components remain the same or grow as the component SFs go up from low to intermediate values, followed by a steep decline when the component SFs increase further (Figures 5C, 5F, and 5I; Figure 6D; Figures 7D to 7F; Figure 8B; and Figure 9C). As Quaia and colleagues noted, a reason underlying certain differences between disparity and motion processing might be “ecological: motion and disparity signals have different patterns of occurrence in the environment” (Quaia, FitzGibbon, Optican, & Cumming, 2019). On the other hand, fitting the responses to the motion of two or three combined gratings versus broadband stimuli had its share of dissimilar best-fit parameters. The differences in the shape of the SF-dependent weight functions were just outlined above. Also, TF-based weight corrections were crucial when fitting gratings data but were not necessary when fitting broadband data. Finally, for both OFRs and DVRs, power summation coefficients (free parameter *m*) were usually higher than 1 for gratings (median, 1.2; range, 1–1.5) and lower than the 1 for white noise stimuli (median, 0.78; range, 0.66–0.99). These grating and white noise discrepancies in the parameters of our model may be due to the fact that the model is descriptive: it operates with OFRs and DVRs to Fourier components of the stimuli, whereas the real-life primary cortical disparity and motion detectors are bandpass. The SF tuning of neurons in striate cortex of primates (De Valois, Albrecht, & Thorell, 1982; Schiller, Finlay, & Volman, 1976) and cats (Kulikowski & Bishop, 1981; Movshon, Thompson, & Tolhurst, 1978) was best described by a fixed bandwidth on a log scale, the median being around 1.4 octaves, decreasing slightly for detectors having higher central SFs (De Valois et al., 1982). Thus, a single detector could be sensitive to a few (or many) Fourier components. When using broadband stimuli, many detectors are concurrently activated whose bandwidths may overlap or not. When using gratings, on the other hand, we usually chose SFs that were far apart; thus, such stimuli activated detectors that had little or no bandwidth overlap. We still have limited knowledge regarding the interactions between different detectors, but they potentially might be the reason for observed variations in best-fit parameters of our model for gratings versus broadband stimuli. We also know that normalization occurs at different stages of processing (retina, lateral geniculate nucleus, V1, MT, etc.). Because of the nonlinearities, cascading multiple normalization stages can give rise to peculiar overall functions, and even relatively small differences at a single stage might result in large differences downstream (for a discussion of these issues, see Quaia et al., 2017b; Quaia et al., 2018).

Shortening the stimulus linear dimensions resulted in a shift of the SF-dependent weight functions toward higher SFs (Figure 3; see also figure 7 in Sheliga et al., 2020) and produced similar shifts in the OFR SF-tuning curves (Figure 4^5^,^6^,^7^,^8^,^9^; see also figure 9 in Sheliga et al., 2020), suggesting that similar circuitry might be responsible for both changes. The shift toward higher SFs was larger when the height of the stimulus was reduced rather than its width—that is, the effect was anisotropic. With our spatially oriented stimuli (sinusoidal gratings and 1D white noise), the effect was greater when the stimulus dimensions were altered in a direction parallel to the oriented contour than orthogonal to it. The anisotropy that we observed might largely reflect the effect of orientation-selective surround inhibition and may therefore share the same mechanism as the surround suppression reported for OFR (Barthelemy, Fleuriet, Perrinet, & Masson, 2022; Barthelemy, Vanzetta, & Masson, 2006; Sheliga, Quaia, Cumming, & Fitzgibbon, 2012). The anisotropy is not evident with two-dimensional white noise stimuli (Sheliga, Quaia, FitzGibbon, & Cumming, 2015), pointing to a cortical origin, and resembles the one reported for striatal neurons stimulated with sinusoidal gratings, as the suppressive interactions in primate V1 were stronger in the direction of the ends than the flanks of an orientation-selective receptive field (Cavanaugh, Bair, & Movshon, 2002).

## Footnotes

1 A lookup table contained 100 randomly generated 1D vertical binary RLS samples.

2 For subject BMS, separate sessions were run in which five central SFs of bandpass- and notch-filtered stimuli were set either to 0.088, 0.177, 0.354, 0.707, and 1.414 cpd or to 0.0625, 0.125, 0.25, 0.5, and 2 cpd. The 512 × 512-pixel white noise condition served as a common condition for the OFR amplitude normalization between these two different types of sessions.
